# Effects of Cocoa Consumption on Cardiometabolic Risk Markers: Meta-Analysis of Randomized Controlled Trials

**DOI:** 10.3390/nu16121919

**Published:** 2024-06-18

**Authors:** Tainah O. P. Arisi, Diego Silveira da Silva, Elana Stein, Camila Weschenfelder, Patrícia Caetano de Oliveira, Aline Marcadenti, Alexandre Machado Lehnen, Gustavo Waclawovsky

**Affiliations:** 1 Instituto de Cardiologia do Rio Grande do Sul/Fundação Universitária de Cardiologia, Porto Alegre 90620-001, RS, Brazil; tainahortiz05@gmail.com (T.O.P.A.); dieguitoef@hotmail.com (D.S.d.S.); elanast.nutricao@gmail.com (E.S.); camilawesche@gmail.com (C.W.); fisio.patriciacaetano@gmail.com (P.C.d.O.); marcadenti@yahoo.com.br (A.M.); gwaclawovsky@gmail.com (G.W.); 2 Instituto de Pesquisa Hcor (IP-Hcor), Hcor, São Paulo 04005-909, SP, Brazil; 3 Faculdade de Saúde Pública, Universidade de São Paulo (FSP-USP), São Paulo 01246-904, SP, Brazil

**Keywords:** cocoa, polyphenols, obesity, dyslipidemia, fasting glucose, blood pressure

## Abstract

Background: We conducted a systematic review and meta-analysis to examine the effect of dietary intake of cocoa on anthropometric measurements, lipid and glycemic profiles, and blood pressure levels in adults, with and without comorbidities. Methods: The databases used were MEDLINE (PubMed), EMBASE, Web of Science, Cochrane, LILACS, and SciELO. The eligible studies were randomized clinical trials (RCTs) involving adults undergoing cocoa consumption (cocoa extract or ≥70% cocoa dark chocolate) for ≥4 weeks that evaluated at least one of the following markers: body weight, body mass index (BMI), waist/abdominal circumference, total cholesterol, LDL-c, triglycerides, HDL-c, blood glucose, glycated hemoglobin (HbA1c), and systolic and diastolic blood pressure (SBP/DBP). Results: Thirty-one studies were included, totaling 1986 participants. Cocoa consumption showed no effects on body weight, BMI, waist circumference, triglycerides, HDL-c and HbA1c. Yet, there was a reduction in total cholesterol (−8.35 mg/dL, 95% CI −14.01; −2.69 mg/dL), LDL-c (−9.47 mg/dL, 95% CI −13.75; −5.20 mg/dL), fasting blood glucose (−4.91 mg/dL, 95% CI −8.29; −1.52 mg/dL), SBP (−2.52 mmHg, 95% CI −4.17; −0.88 mmHg), and DBP (−1.58 mmHg, 95% CI −2.54; −0.62 mmHg). Conclusions: The consumption of cocoa showed protective effects on major cardiometabolic risk markers that have a clinical impact in terms of cardiovascular risk reduction.

## 1. Introduction

Cardiovascular diseases (CVDs), whether or not associated with adverse metabolic risk factors, accounted for 397,000 deaths in Brazil in 2019 [1]. Coronary artery disease is strongly associated with atherosclerosis and dyslipidemia and accounted for 171,246 deaths (43% of total CVD deaths). In addition, there were 80,754 deaths from diabetes mellitus (20%) that same year [1]. Interestingly, since the epidemiological “black box” concept has been introduced in the mid-1940s, we have sought to understand the relationship of CVD risk factors associated with adverse metabolic conditions, such as central obesity, type 2 diabetes mellitus, and dyslipidemia, among others. The Framingham Heart Study [2] was a pioneer and is the basis for the current cardiometabolic risk stratification [3] that includes age; sex; body weight-to-height ratio (body mass index, BMI); systolic blood pressure (SBP) and diastolic blood pressure (DBP) levels or well-established systemic arterial hypertension; fasting blood glucose or well-established type 2 diabetes mellitus; total cholesterol; high-density lipoprotein cholesterol (HDL-c); and smoking.

Scientific research studies have been investigating robust interventions to address these markers and reduce the risk and/or minimize the burden of cardiometabolic diseases. Some of these interventions involve nutritionally adequate diets that may have a positive impact on total body weight [4] and/or BMI, waist/abdominal circumference as a marker of central obesity, lipid profile [5], glycemic profile [6], and BP levels [7], among others. In particular, the consumption of foods containing bioactive substances, such as polyphenols [8], has been associated with improvements in major predictors of CVDs [9,10].

*Theobroma cacao*, popularly known as cocoa, is a fruit rich in polyphenols, mostly flavonoids [11,12]. Cocoa might exert beneficial cardiovascular effects that are probably mediated by this group of molecules. A range of potential mechanisms through which cocoa might improve cardiovascular health have been suggested, including the activation of nitric oxide (NO) and antioxidant/anti-inflammatory effects. This may explain the positive effects on endothelial function and the reduction of platelet function, blood pressure, markers of insulin resistance, and blood lipids found in epidemiological studies [13]. However, the impact of cocoa consumption in reducing cardiometabolic risk markers (listed in the Framingham risk score) has yet to be established, since the results from randomized clinical trials (RCTs) are controversial. In addition, the amount, frequency, and form of both cocoa and polyphenol intake that could provide these benefits has not been established.

We conducted a systematic review and meta-analysis of RCTs to examine the long-term effects of dietary cocoa consumption on cardiometabolic risk markers, including anthropometric measurements, lipid profile, and blood pressure levels, in adults with and without established comorbidities. Our initial hypothesis was that cocoa has adequate phenolic properties to exert cardioprotective effects on selected outcomes of interest, such as total body weight; BMI; waist/abdominal circumference; total cholesterol; low-density lipoprotein cholesterol (LDL-c); triglycerides; HDL-c; fasting blood glucose and glycated hemoglobin (HbA1c); and SBP and DBP levels.

## 2. Materials and Methods

**Figure 1:**
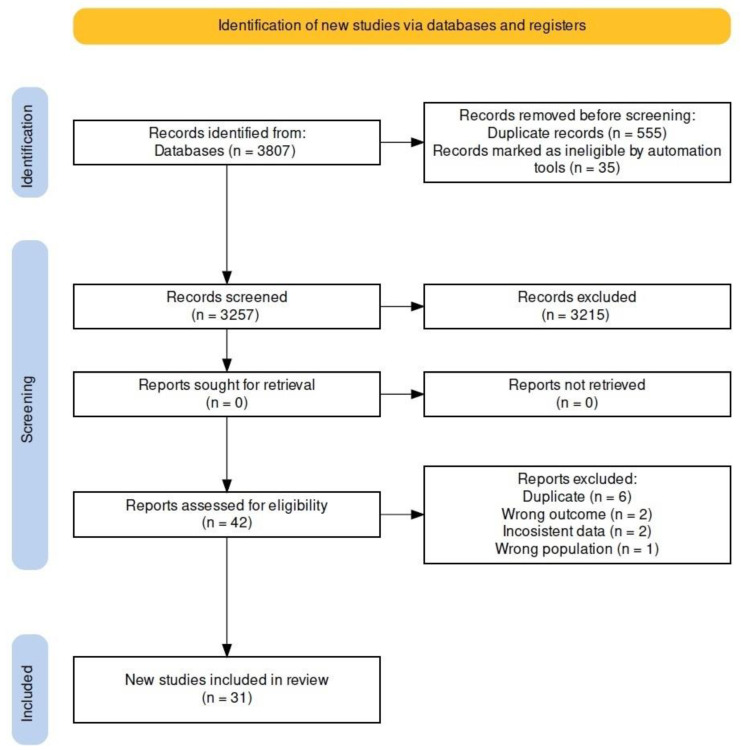
Flowchart of the study selection process.

The study protocol was developed based on PRISMA (preferred reporting items for systematic review and meta-analysis) recommendations [14] and the Cochrane methodology for systematic reviews [15]. The protocol for this systematic review and meta-analysis was registered in the International Prospective Register of Systematic Reviews (PROSPERO) (www.crd.york.ac.uk/PROSPERO/, 17 May 2024) and ID “CRD42023484490”, 30 November 2023. The databases and script (Rstudio, version 1.3.959, and R package meta for Windows, version 3.6.1) used in this systematic review for the metanalysis are available on the Mendeley Data repository as open access (https://data.mendeley.com/; doi:10.17632/mwtwh6d8ws.1, 17 May 2024). Figure 1 summarizes the flowchart of the study design.

### 2.1. Eligibility Criteria

We used the PICOS design as a framework to formulate eligibility criteria as follows: population: age ≥18 years, healthy or diagnosed with arterial hypertension, and/or type 2 diabetes mellitus and/or dyslipidemia and/or overweight/obesity and/or myocardial infarction/stroke; intervention: cocoa-extract supplement or ≥70% cocoa dark chocolate for ≥4 weeks; comparison: placebo or <70% cocoa white/milk chocolate; outcomes: body weight (kg), BMI (kg/m^2^), or waist or abdominal circumference (cm) as an indicator of central obesity; total cholesterol (mg/dL), LDL-c (mg/dL), triglycerides (mg/dL), HDL-c (mg/dL); fasting blood glucose (mg/dL) and HbA1c (%); and SBP and DBP (mmHg); study: RCTs only.

We chose to examine adult populations with or without established cardiometabolic risk factors (arterial hypertension and/or type 2 diabetes mellitus and/or dyslipidemia and/or overweight/obesity and/or myocardial infarction/stroke), as their characteristics are closely associated with our research question.

As for the intervention, cocoa can be consumed in multiple variations without restrictions. Still, there is no clear definition of dark chocolate and its cocoa content. We used the United States Department of Agriculture (USDA) food data to standardize chocolate with high cocoa content (>70%) (https://fdc.nal.usda.gov/fdc-app.html#/food-details/170273/nutrients, accessed on 4 June 2024), low content (control) (https://fdc.nal.usda.gov/fdc-app.html#/food-details/170271/nutrients, accessed on 4 June 2024), and “cocoa, dry powder, unsweetened” (https://fdc.nal.usda.gov/fdc-app.html#/food-details/169593/nutrients, accessed on 4 June 2024).

In addition, we performed subgroup analyses comparing healthy and non-healthy individuals with cardiometabolic risk factors and discussed the results. For subgroup analyses by daily amount of polyphenol intake, we selected only RCTs reporting the amount of polyphenol intake in both the intervention and the control groups, and we calculated the median for each outcome of interest. After that, the results were then stratified as ‘below’ and ‘above’ the median (Supplementary Material File S3, Figures S11–S20). The authors were contacted by email to obtain any additional information not available and asked to respond within 15 days (maximum contact attempts of three).

### 2.2. Inclusion and Exclusion Criteria

Studies combining other dietary interventions with a well-defined intervention group of cocoa or dark-chocolate intake and a control group were carefully reviewed for inclusion. Studies with participants receiving medications or diet changes were eligible when they were introduced at least four weeks prior to the start of the intervention or were consistent throughout the study to allow for accurate analyses.

We excluded studies with participants undergoing treatments other than for cardiometabolic conditions; pregnant and post-menopausal women; concomitant use of dietary supplements that were not clearly different from the cocoa intervention; review or protocol studies; animal experimentation studies; and studies of conditions other than those related to cardiometabolic health such as cancer. Studies involving the same sample published in different journals were thoroughly reviewed, and the authors were contacted by email for additional information and asked to reply within 15 days (maximum contact attempts of three) and excluded if duplicates.

### 2.3. Search Strategy

Our search strategy for RCTs was developed and conducted by four independent reviewers (two pairs) in the databases recommended in the *Cochrane Handbook for Systematic Reviews of Interventions* [15]: MEDLINE (PubMed), EMBASE (European literature), Web of Science, and Cochrane (for trials that were not indexed in MEDLINE and EMBASE). To broaden our search, we also carried out searches of publications from Latin America in LILACS (Latin American and Caribbean Health Sciences Literature/Virtual Health Library [VHL]) and SciELO (Scientific Electronic Library Online). For unpublished ongoing studies, we searched the following clinical trial registries: ClinicalTrial.gov; the Brazilian Clinical Trials Registry (REBEC); and the World Health Organization (WHO) International Clinical Trials Registry Platform (ICTRP). We also searched preprint databases (preprints.org/, biorxiv.org, and medrxiv.org).

Articles in the Portuguese, English, and Spanish languages with no date-of-publication limits were eligible for inclusion. Upon completion of the review, we performed an additional search of all databases and registry platforms to ensure the inclusion of the most recent studies.

The main search terms included ‘cocoa’, ‘Theobroma cacao’, and ‘dark chocolate’ (Table S1, Supplementary Material File S1). To increase the accuracy and sensitivity of our searches, the search terms for the study design (RCT) were entered into the databases MEDLINE [16] and EMBASE [17] (Table S1, Supplementary Material File S1). Studies were independently selected and assigned to four reviewers after an initial screening of titles and abstracts. When abstracts did not contain enough information, the reviewers read the full text of the article. Any disagreements among the reviewers were resolved through discussion and, if no consensus was reached, a fifth reviewer (WG) was consulted.

### 2.4. Data Extraction and Management

After completing our searches in each database, all articles retrieved were exported as .ris or .enib files and imported into the Rayyan reference manager [18]. Duplicate studies were removed; our blinded reviewers manually checked for the remaining duplicates. They used the Rayyan application “to include” articles that met the eligibility criteria. Those that did not meet the inclusion criteria were marked as “excluded” and categorized by reason for exclusion (e.g., ineligible outcomes or population; non-RCT design). When it was not clear whether a study should be included or excluded, it was marked as “undecided”. In a virtual meeting, a pair of reviewers expressed their concerns regarding the “inclusion” or “exclusion” of an article to the other two reviewers. The issues were discussed among them and, if there was no consensus, they were presented to and resolved by a fifth reviewer (WG).

The four blinded reviewers independently performed data extraction. They used a pre-structured Excel 2019 database divided into columns to compile the data extracted, including reviewer; author; year; journal; country; inclusion and exclusion criteria; analysis strategy; the number of individuals included and excluded; the number of individuals analyzed; sex; age; follow-up period; nutritional supplement taken; how it was supplemented; daily amount; and risk of bias. For the main analysis, baseline and post-intervention data were extracted (mean and dispersion measures) for the following markers: body weight, BMI, waist and abdominal circumference; total cholesterol, LDL-c, triglycerides and HDL-c; blood glucose, HbA1c; and SBP and DBP. For the extraction of data from eligible studies with the results presented in graphs, we contacted the authors by email to obtain these data or used GetDate Graph Digitizer 2.26 to extract them.

### 2.5. Risk of Bias and Strength of Evidence

The risk of bias of RCTs was assessed using the Cochrane risk of bias (RoB) 2 tool included in the *Cochrane Handbook* [15,19,20]. The analysis was based on a set of six domains of bias and rated as low, high, or unclear risk of bias: randomization sequence generation, allocation concealment, blinding of participants and personnel, blinding of outcome assessment, incomplete outcome data, and selective reporting. When participants were not blinded, studies were classified as high risk of bias in the “blinding of participants and personnel” domain.

We assessed the strength of evidence using the Grading of Recommendations Assessment, Development, and Evaluation (GRADE) tool [21,22]. This tool assesses confidence in paired-effect estimates and classifies treatment effect in a meta-analysis (high, moderate, low, and very low confidence) in the following domains [21,22]: study design, methodological limitations (risk of bias), inconsistency, indirectness of evidence, imprecision, publication bias, magnitude of effect, dose-response gradient, and residual confounders.

GRADE assessments were conducted in a separate step upon the completion of data extraction. Since our meta-analysis included RCTs only, the recommendation is first to rate the study design as “high” [21]. As for the risk of methodological bias, we read the full text of all the articles included and rated them as “low risk”, “moderate risk”, or “high risk” for each item as follows: random sequence generation, allocation concealment, blinding (performance, participants and outcome assessment), incomplete outcome data, selective reporting, and other bias). A score was then generated for methodological bias. The item “inconsistency” was evaluated by the similarity of effect estimates with a 95% CI overlapping, as well as the degree of heterogeneity (I^2^); “indirectness of evidence” considers similarities between the participants, interventions, and outcomes evaluated. In our meta-analysis, they were all rated as “no risk” because of similar inclusion/exclusion criteria. Using forest plots with wide 95% CIs for each study and outcome, “imprecision” was assessed visually. The “risk of publication bias” was assessed by the symmetry of the funnel plot (Supplementary Material File S4, Figures S21–S30); “magnitude of effect, dose-response gradient, and residual confounders” would not imply a decrease of the strength of evidence, but rather would increase the strength of evidence, especially in studies with relative risk estimates.

### 2.6. Data-Analysis Strategy

We performed statistical analyses to estimate the effects of cocoa and/or ≥70% cocoa dark-chocolate consumption on anthropometric measurements, lipid and glycemic profiles, and BP levels compared to a control group. Summary effect estimates were expressed in terms of mean difference (MD) and 95% confidence interval (95% CI) pooled using a random-effects model and inverse variance method. We considered the calculated values for a prediction interval (PI), as they reflect the interval of uncertainty of the effects to be expected in future RCTs [23]. To avoid unit-of-analysis errors for RCTs with multiple treatment arms and a single control group, the number of participants in the control group was weighted by the number of groups and participants undergoing the intervention.

To assess the consistency of cocoa’s and/or ≥70% cocoa dark chocolate’s effects across studies, the degree of heterogeneity was tested using the inconsistency test by Higgins (*I^2^*) for every pairwise comparison [15,24]. To explore heterogeneity, we performed subgroup analyses and meta-regression analyses (≥10 studies) for effect modifiers with normal distribution in a quartile-quartile plot (qq-plot) and confirmed it using the Shapiro–Wilk test (*p* > 0.05) [25]. Non-normal data were normalized before performing the meta-regression analysis. When applicable (≥10 studies; more than one study with significant statistical data; studies with different sample sizes), we performed Egger’s test using a funnel plot to assess the potential publication bias in the meta-analysis [26,27].

Dispersion measures expressed as confidence intervals (CI) or standard errors (SE) were converted to standard deviation (SD = EP × √n) before the analysis. For eligible studies that did not report the SD of differences, the SD was estimated using an imputed correlation coefficient (CC) of 0.5, as described in Section 6.5.2.8, *Cochrane Handbook* [28]: Δ SD = √ SD^2^ baseline + SD^2^ final − (2 × CC × SD baseline × SD final). Two-tailed tests were used at a significance level of *p* < 0.05. Finally, we used RStudio (version 1.3.959) with the R package meta (version 3.6.1) for Windows.

## 3. Results

### 3.1. Characteristics of the Studies

Table 1 summarizes the characteristics of the studies selected for this review. The final analysis involved 31 studies [29,30,31,32,33,34,35,36,37,38,39,40,41,42,43,44,45,46,47,48,49,50,51,52,53,54,55,56,57,58,59] totaling 1986 participants, i.e., 1110 in the intervention group and 876 in the control group. The clinical characteristics of the participants were individually defined, but most studies involved participants with more than one health condition. Briefly, 13 studies involved healthy participants [30,31,33,37,39,41,45,47,48,49,57,59]; three with metabolic syndrome [29,54,58]; four with dyslipidemia [36,37,51,55]; two with pre-hypertension and/or hypertension [42,43]; four with excess weight [40,44,46,51]; seven with type 2 diabetes mellitus [32,35,42,43,52,53,56]; and one with insulin resistance [50]. Four studies evaluated older participants only [38,39,45,57] and five young individuals only [30,33,41,58,59]. 

We conducted a thorough search of the literature, including the following databases: ClinicalTrials.gov (www.clinicaltrials.gov, accessed on 2 April 2024); Preprints (www.preprints.org, accessed on 2 April 2024); OpenGrey (www.opengrey.eu, accessed on 2 April 2024); the Brazilian Coordination for the Improvement of Higher Education Personnel (CAPES) Bank of Theses and Dissertations; and Brazilian Clinical Trials Registry (REBEC) (https://ensaiosclinicos.gov.br/, accessed on 2 April 2024). A total of 76 articles were retrieved from the ClinicalTrials.gov database, but they were all studies already retrieved in our initial search. We found no articles in Preprints and two articles in OpenGrey, but they were not eligible for inclusion in the review as they did not include outcomes of interest. We retrieved from the CAPES database two master dissertations based on a similar research topic and outcomes, but they were not eligible for inclusion due to sample overlap and no control group. Articles in the Portuguese, English, and Spanish languages with no date-of-publication limits were eligible for inclusion, and no articles were found in the REBEC database.

The mean duration of the intervention was 12 weeks (range 4–24 weeks), and the daily amount of cocoa intake ranged from 1.4 to 50.0 g. The types of bioactive compounds and their contents varied (Table 1). The types of cocoa products consumed included capsules (three studies) [31,32,47]; cocoa extracts and beverages (17 studies) [29,35,36,37,38,39,40,44,45,46,48,49,52,53,54,56,57]; cocoa bars/dark chocolate (nine studies) [30,33,41,42,43,50,57,58,59]; cocoa snacks (two studies) [51,55]; and mixed in the meal ingredients (one study) [34].

### 3.2. Meta-Analysis of the Effect on Anthropometric Measurements

**Figure 2:**
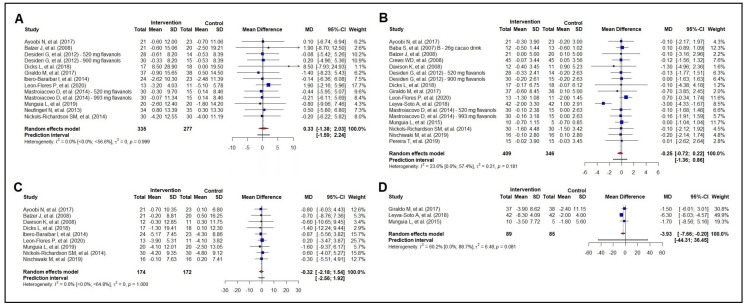
Forest plot summarizing the results from the meta-analysis regarding the effects of cocoa consumption on anthropometric measurements versus control group. Panel (**A**): body weight: Ayoobi et al. 2017 [35]; Balzer et al. 2008 [53]; Desideri et al. 2012 [38]; Dicks et al. 2018 [32]; Giraldo et al. 2017 [50]; Ibero-Baraibar et al. 2014 [34]; León-Flores et al. 2020 [51]; Mastroiacovo et al. 2015 [39]; Munguia et al. 2019 [45]; Neufingerl et al 2013 [49]; Nickols-Richardson et al. 2014 [46]. panel (**B**): body mass index: Ayoobi et al. 2017 [35]; Baba et al. 2007 [37]; Balzer et al. 2008 [53]; Crews et al. 2008 [57]; Davison et al. 2008 [40]; Desideri et al. 2012 [38]; Dicks et al. 2018 [32]; Giraldo et al. 2017 [50]; León-Flores et al. 2020 [51]; Leyva-Soto et al. 2018 [58]; Mastroiacovo et al. 2015 [39]; Munguía et al. 2015 [44]; Nickols-Richardson et al. 2014 [46]; Nishiwaki et al. 2019 [41]; Pereira et al. 2019 [33]. panel (**C**): waist circumference: Ayoobi et al. 2017 [35]; Balzer et al. 2008 [53]; Davison et al. 2008 [40]; Dicks et al. 2018 [32]; Ibero-Baraibar et al. 2014 [34]; León-Flores et al. 2020 [51]; Munguia et al. 2019 [45]; Nickols-Richardson et al. 2014 [46]; Nishiwaki et al. 2019 [41]. panel (**D**): abdominal circumference: Giraldo et al. 2017 [50]; Leyva-Soto et al. 2018 [58]; Munguía et al. 2015 [44].

The effect of cocoa consumption on total body mass was evaluated in 11 studies (n = 612) and was found to be ineffective (Figure 2A), even in the subgroup analysis (Figure S1, Supplementary Material File S2; Figure S11, Supplementary Material File S3). The analysis of BMI involved 15 studies (n = 755), and we found no effect of cocoa consumption on reducing BMI; the subgroup analysis did not show any differences (Figure S2, Supplementary Material File S2; Figure S12, Supplementary Material File S3). The same was seen for waist circumference (nine studies; n = 346), as well as in the subgroup analysis (Figure S3, Supplementary Material File S2; Figure S13, Supplementary Material File S3).

As for abdominal circumference (three studies; n = 174), our meta-analysis showed a reduction in abdominal circumference by 3.93 cm (95% CI −7.66 to −0.20 cm) (Figure 2D). However, there was moderate heterogeneity across the studies [60.2% (0.0%; 88.7%)]. Two studies coincided with the null line, and one study showed a favorable effect of the cocoa intervention.

Meta-regressions analyzing the potential effect modifiers, including the baseline total body mass, baseline waist circumference, baseline BMI, age, number of interventions, and amount of polyphenol intake, found no effect for the variables of total body mass, waist circumference, and abdominal circumference. However, in the intervention group, the baseline BMI proved to be an effect modifier, with an effect modification of 77.88% (*p* = 0.034). For every increase in baseline BMI of 1.0 kg/m^2^, there was a reduction of 0.14 kg/m^2^ following the cocoa intervention. A sensitivity analysis for the anthropometric measurements is available in Supplementary Material File S5.

### 3.3. Meta-Analysis of the Effect on Lipid Profile

**Figure 3:**
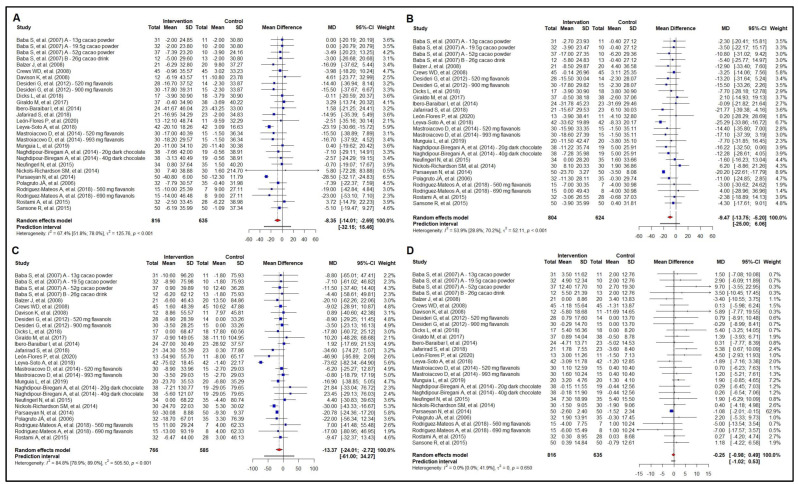
Forest plot summarizing the results from the meta-analysis regarding the effects of cocoa consumption on lipid profile versus control group. Panel (**A**): total cholesterol; panel (**B**): low-density lipoprotein cholesterol (LDL-c). Baba et al. 2007 [37]; Baba et al. 2007 [36]; Balzer et al. 2008 [53]; Crews et al. 2008 [57]; Desideri et al. 2012 [38]; Dicks et al. 2018 [32]; Giraldo et al. 2017 [50]; Ibero-Baraibar et al. 2014 [34]; Jafarirad et al. 2018 [52]; León-Flores et al. 2020 [51]; Leyva-Soto et al. 2018 [58]; Mastroiacovo et al. 2015 [39]; Munguia et al. 2019 [45]; Naghdipour-Biregani et al. 2014 [54]; Neufingerl et al 2013 [49]; Nickols-Richardson et al. 2014 [46]; Parsaeyan et al. 2014 [56]; Polagruto et al. 2006 [55]; Rodriguez-Mateos et al. 2018 [47]; Rostami et al. 2015 [42]; Sansone et al. 2015 [48]. panel (**C**): triglycerides; Baba et al. 2007 [37]; Baba et al. 2007 [36]; Balzer et al. 2008 [53]; Crews et al. 2008 [57]; Davison et al. 2008 [40]; Desideri et al. 2012 [38]; Dicks et al. 2018 [32]; Giraldo et al. 2017 [50]; Ibero-Baraibar et al. 2014 [34]; Jafarirad et al. 2018 [52]; León-Flores et al. 2020 [51]; Leyva-Soto et al. 2018 [58]; Mastroiacovo et al. 2015 [39]; Munguia et al. 2019 [45]; Naghdipour-Biregani et al. 2014 [54]; Neufingerl et al 2013 [49]; Nickols-Richardson et al. 2014 [46]; Parsaeyan et al. 2014 [56]; Polagruto et al. 2006 [55]; Rodriguez-Mateos et al. 2018 [47]; Rostami et al. 2015 [42]; and panel (**D**): high-density lipoprotein cholesterol (HDL-c). Panel (**A**,**D**): Baba et al. 2007 [37]; Baba et al. 2007 [36]; Balzer et al. 2008 [53]; Crews et al. 2008 [57]; Davison et al. 2008 [40]; Desideri et al. 2012 [38]; Dicks et al. 2018 [32]; Giraldo et al. 2017 [50]; Ibero-Baraibar et al. 2014 [34]; Jafarirad et al. 2018 [52]; León-Flores et al. 2020 [51]; Leyva-Soto et al. 2018 [58]; Mastroiacovo et al. 2015 [39]; Munguia et al. 2019 [45]; Naghdipour-Biregani et al. 2014 [54]; Neufingerl et al 2013 [49]; Nickols-Richardson et al. 2014 [46]; Parsaeyan et al. 2014 [56]; Polagruto et al. 2006 [55]; Rodriguez-Mateos et al. 2018 [47]; Rostami et al. 2015 [42]; Sansone et al. 2015 [48].

Twenty-two studies examined the effect of cocoa consumption on total cholesterol (n = 1451), evaluating a wide range of cocoa percentages. Dietary cocoa consumption effectively reduced total cholesterol by −8.35 mg/dL (95% CI −14.01 to −2.69 mg/dL) (Figure 3A). Interestingly, the subgroup analysis by health status (healthy vs. unhealthy) (Figure S4, Supplementary Material File S2) showed a reduction in total cholesterol of −7.11 mg/dL (95% CI −12.13 to −2.09 mg/dL) in unhealthy participants and of −7.37 mg/dL (95% CI −15.38 to 0.63 mg/dL) in healthy participants, though with no difference in the effect size between these groups (*p* = 0.959). Furthermore, this effect seems to be associated with a daily polyphenol intake above the median (369.7 mg) found in our meta-analysis (Figure S14, Supplementary Material File S3).

The meta-analysis of the effect of cocoa consumption on LDL-c included the same above-mentioned studies that evaluated total cholesterol. However, the [51,55] study was excluded from this analysis, as we detected outlier values for LDL-c [40] in the sensitivity analysis and found a non-overlapping 95% CI with other studies. The analysis showed a reduction in LDL-c of −9.47 mg/dL (95% CI −13.75 to −5.20) with cocoa consumption (Figure 3B). The sub-analyses by health status (healthy vs. unhealthy) showed a reduction in LDL-c of −8.44 mg/dL (95% CI −13.03 to −3.85) only among the participants with unhealthy status (Figure S5, Supplementary Material File S2). The beneficial effects on LDL-c seem to be independent of the daily amount of polyphenol intake (Figure S15, Supplementary Material File S3).

Figure 3C shows the results of the meta-analysis of the effect of cocoa consumption on triglycerides. The effect size in reducing triglycerides was −13.37 mg/dL (95% CI −24.01 to −2.72 mg/dL). We also carried out subgroup analyses. Interestingly, the effect of cocoa consumption (Figure S6, Supplementary Material File S2) and daily polyphenol intake (Figure S16, Supplementary Material File S3) was no longer seen in both unhealthy and healthy participants.

Finally, as for the effect of cocoa consumption on HDL-c (Figure 3D), our analysis included the same studies as mentioned above for total cholesterol and LDL-c. Yet, it showed no effect on HDL-c. The same was seen in the subgroup analyses by health status (unhealthy vs. healthy) (Figure S7, Supplementary Material File S2) and daily amount of polyphenol intake (Figure S17, Supplementary Material File S3).

Meta-regressions analyzing potential effect modifiers (age, number of interventions, amount of polyphenol intake and baseline values of total body mass, total cholesterol, LDL-c, HDL-c, triglycerides, and BMI) were not found for the variables of total cholesterol, LDL-c, and HDL-c. However, for triglycerides, baseline BMI in the intervention group (61.82%; *p* = 0.025) and age in the intervention group (77.58%; *p* = 0.001) and control group (78.10%; *p* = 0.001) proved to be a strong effect modifier. In addition, we found a reduction in triglycerides of 3.06 mg/dL in the intervention group for every increase in baseline BMI of 1.0 kg/m^2^. As for age, in both the intervention and control groups, the data showed an increase in triglycerides of 0.84 mg/dL for every one-year increase in the participants’ age. A sensitivity analysis for the lipid profile is available in Supplementary Material File S5.

### 3.4. Meta-Analysis of the Effect on Glycemic Profile

**Figure 4:**
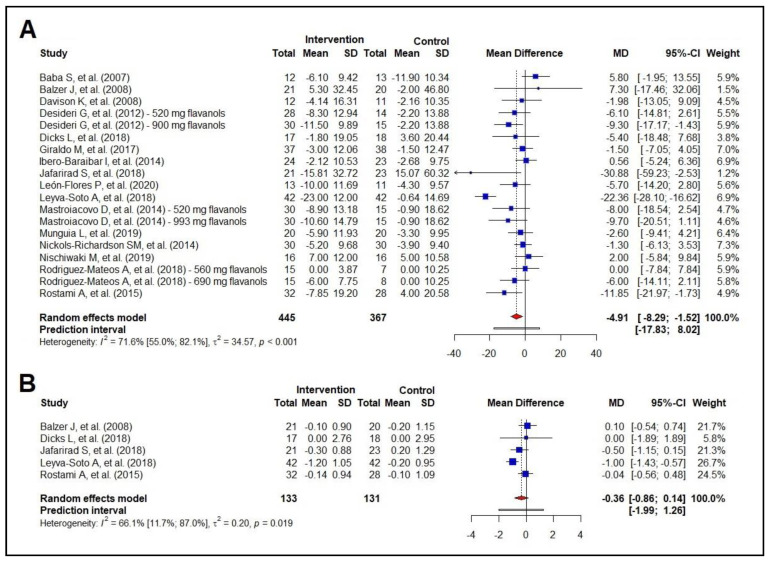
Forest plot summarizing the results from the meta-analysis regarding the effects of cocoa consumption on glycemic markers versus control group. Panel (**A**): fasting blood glucose, Baba et al. 2007 [37]; Balzer et al. 2008 [53]; Davison et al. 2008 [40]; Desideri et al. 2012 [38]; Dicks et al. 2018 [32]; Giraldo et al. 2017 [50]; Ibero-Baraibar et al. 2014 [34]; Jafarirad et al. 2018 [52]; León-Flores et al. 2020 [51]; Leyva-Soto et al. 2018 [58]; Mastroiacovo et al. 2015 [39]; Munguia et al. 2019 [45]; Nickols-Richardson et al. 2014 [46]; Nishiwaki et al. 2019 [41]; Rodriguez-Mateos et al. 2018 [47]; Rostami et al. 2015 [42]; panel (**B**): glycated hemoglobin. Balzer et al. 2008 [53]; Dicks et al. 2018 [32]; Jafarirad et al. 2018 [52]; Leyva-Soto et al. 2018 [58]; Rostami et al. 2015 [42].

The results showed a reduction of −4.91 mg/dL in blood glucose in response to cocoa consumption (95% CI −8.29 to −1.52 mg/dL) (Figure 4A). Cocoa consumption led to a reduction in fasting blood glucose of −4.57 mg/dL (95% CI −8.37 to −0.77) only among the participants with an unhealthy status (Figure S8, Supplementary Material File S2). It is worth noting that this effect seems to be significant when the polyphenol intake is above the median (479.0 mg) (Figure S18, Supplementary Material File S3).

The other outcome of interest in glucose metabolism was HbA1c, and the results showed no effect of cocoa consumption on HbA1c (Figure 4B). Considering a borderline significance and the small number of studies evaluating HbA1c, further investigations may reveal a trend and help test new hypotheses about the effects of cocoa consumption on glucose metabolism.

Meta-regressions analyzing potential effect modifiers, including baseline total body mass, baseline fasting blood glucose, baseline BMI, age, number of interventions, and amount of polyphenol intake did not show these variables to be effect modifiers for fasting blood glucose. Due to the small number of studies evaluating the effect of cocoa on HbA1c, we could not perform a meta-regression. A sensitivity analysis for fasting blood glucose and HbA1c is available in Supplementary Material File S5.

### 3.5. Meta-Analysis of the Effect on Blood Pressure

**Figure 5:**
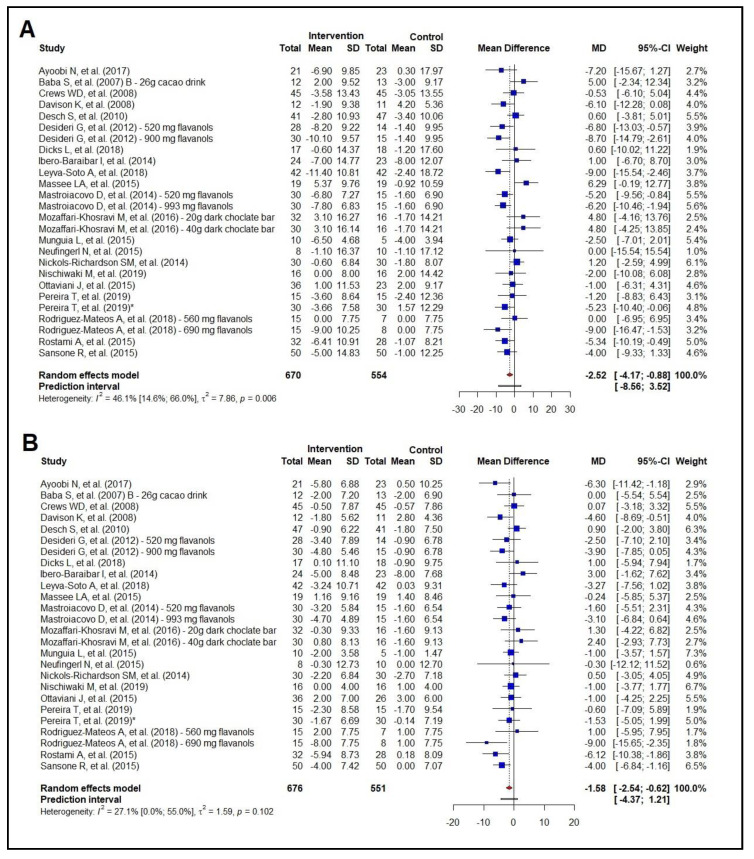
Forest plot summarizing the results from the meta-analysis regarding the effects of cocoa consumption on blood pressure versus control group. Panel (**A**): systolic blood pressure. Ayoobi et al. 2017 [35]; Baba et al. 2007 [37]—26 g; Crews et al. 2008 [57]; Davison et al. 2008 [40]; Desch et al. 2010 [43]; Desideri et al. 2012 [38]; Dicks et al. 2018 [32]; Ibero-Baraibar et al. 2014 [34]; Leyva-Soto et al. 2018 [58]; Massee et al. 2015 [30]; Mastroiacovo et al. 2015 [39]; Mozaffari-Khosravi et al. 2016 [29]; Munguía et al. 2015 [44]; Neufingerl et al 2013 [49]; Nickols-Richardson et al. 2014 [46]; Nishiwaki et al. 2019 [41]; Ottaviani et al. 2015 [31]; Pereira et al. 2019 [33]; Rodriguez-Mateos et al. 2018 [47]; Rostami et al. 2015 [42]; Sansone et al. 2015 [48]; panel (**B**): diastolic blood pressure. Ayoobi et al. 2017 [35]; Baba et al. 2007 [37]—26 g; Crews et al. 2008 [57]; Davison et al. 2008 [40]; Desch et al. 2010 [43]; Desideri et al. 2012 [38]; Dicks et al. 2018 [32]; Ibero-Baraibar et al. 2014 [34]; Leyva-Soto et al. 2018 [58]; Massee et al. 2015 [30]; Mastroiacovo et al. 2015 [39]; Mozaffari-Khosravi et al. 2016 [29]; Munguía et al. 2015 [44]; Neufingerl et al 2013 [49].

The overall analysis of the effect of cocoa consumption on blood pressure involved 22 studies (n = 1224) and showed a reduction in SBP of −2.52 mmHg (95% CI −4.17 to −0.88 mmHg) and a reduction in DBP of −1.58 mmHg (95% CI −2.54 to −0.62 mmHg) (Figure 5A,B). The subgroup analyses by health status showed a reduction in SBP of −2.72 mmHg (95% CI −5.05 to −0.40) in the unhealthy group but no effect in the healthy group (MD −1.51 mmHg; 95% CI −4.29 to 1.26) (Figure S9, Supplementary Material File S2). In addition, they showed no effect on DBP in the unhealthy group (MD −1.41 mmHg; 95% CI −2.99 to 0.18), but a reduction in DBP of −1.59 mmHg (95% CI −2.81 to −0.37 mmHg) in the healthy group (Figure S10, Supplementary Material File S2).

The lowering of BP levels seems to be dependent upon the amount of polyphenol intake, as reductions in SBP and DBP were seen only with a daily polyphenol intake above the median (432.2 mg/day) (Figures S19 and S20, Supplementary Material File S3).

Meta-regressions were performed to analyze the potential effect modifiers (baseline body mass, baseline BMI, baseline waist circumference, baseline SBP/DBP, age, number of interventions, and amount of polyphenol intake) for BP levels. For SBP, only the baseline SBP values in the intervention group (72.47%; *p* = 0.001) and the control group (48.18%; *p* = 0.007) proved to be an effect modifier. For every 1.0 mmHg increase in baseline SBP, there was a reduction of 0.27 mmHg in the intervention group and 0.24 mmHg in the control group. We did not find an effect modification for DBP. A sensitivity analysis for BP is available in the Supplementary Material File S5.

### 3.6. Risk of Bias Assessment (RoB 2) and Strength of Evidence (GRADE)

A risk of bias assessment using the RoB 2 tool showed that all RCTs reported randomization [29,30,31,32,33,34,35,36,37,38,39,40,41,42,43,44,45,46,47,48,49,50,51,52,53,54,55,56,57,58,59] of the intervention. Of a total of 31 RCTs included in this meta-analysis, 20 reported blinded the evaluators and participants [30,31,32,33,34,36,37,38,39,40,42,44,45,47,48,49,50,55,57,58], four blinded the evaluators only [35,43,52,56], one blinded the participants only [51], two reported no blinding [41,59] and four did not report the blinding process [36,46,54,56].

Of the 31 studies included in this meta-analysis, nine showed a low risk of bias [17,23,34,40,52,60,61,62,63], as they met the RoB 2 criteria, including prior registration of studies on clinical trial registry platforms. The remaining 22 articles showed some concerns of bias, mostly because they did not present consistent data, especially in the reporting of outcomes domain (“Selection of the reported results”, Figure S31, Supplementary Material File S4).

GRADE was used to assess the strength of the evidence [64]. We rated as “moderate quality” outcomes related to anthropometric measurements (total body mass, BMI, waist circumference, and abdominal circumference) (Table S2, Supplementary Material File S4), lipid profile (total cholesterol, LDL-c, triglycerides, and HDL-c) (Table S3, Supplementary Material File S4), glycemic profile (fasting blood glucose and HbA1c) (Table S4, Supplementary Material File S4), and SBP and DBP (Table S5, Supplementary Material File S4). We chose to downgrade these outcomes by one point in the imprecision domain due to the different characteristics of the samples evaluated and wide confidence intervals. Though there was moderate heterogeneity in the results for some outcomes, we were able to conduct subgroup analyses, meta-regression analysis, and risk of publication bias assessment and, thus, did not rate down the quality of evidence due to inconsistency (heterogeneity), as recommended in the literature [15,25].

## 4. Discussion

We conducted a meta-analysis of RCTs to examine the long-term effects of cocoa consumption on cardiometabolic risk markers, including anthropometric measurements (total body weight, BMI, and waist and abdominal circumference), lipid profile (total cholesterol, LDL-c, triglycerides, and HDL-c), fasting blood glucose and HbA1c, and BP levels (SBP and DBP). The main findings of our study include: (i) cocoa consumption did not consistently reduce body weight, BMI, or waist circumference; (ii) on the other hand, cocoa polyphenol intake reduced total cholesterol (−8.35 mg/dL) and LDL-c (−9.47 mg/dL), but it did not reduce triglycerides and HDL-c; (iii) cocoa consumption was associated with a reduction in fasting blood glucose (−4.91 mg/dL), but not in HbA1c; and (iv) our meta-analysis showed that cocoa consumption reduced both SBP (−2.52 mmHg) and DBP levels (−1.58 mmHg), especially at higher amounts of polyphenol intake. In summary, cocoa consumption showed a protective effect on major cardiometabolic risk markers with a clinically significant impact in terms of cardiovascular risk reduction.

Concerning anthropometric measurements, catechins are the main type of compounds present in cocoa that apparently promote favorable effects on body composition. In addition, cocoa consumption seems to be associated with improved insulin sensitivity in animal models [60], but the findings in the literature are inconsistent in humans. A review of 35 RCTs examined the effects of cocoa and dark-chocolate consumption on total body weight, BMI, and waist circumference [65]. The meta-analysis showed no favorable effects of cocoa supplementation on body weight (MD −0.108 kg; 95% CI −0.262 to 0.046 kg), BMI (MD −0.014 kg/m^2^; 95% CI −0.105 to 0.077 kg/m^2^), and waist circumference (0.025 cm; 95% CI −0.083 to 0.129 cm) [65]. In contrast, an RCT evaluating a regular dietary intake of 99% cocoa dark chocolate (10 g; 64.5 mg of polyphenols) in 132 postmenopausal women for six months reported reductions in both total body weight (−0.63 kg; 95% CI −1.15 to −0.11 kg) and relative fat (−0.79%; 95% CI −1.31 to −0.26%), but no effect on BMI [66]. Despite these favorable results, the effect size was considered not clinically significant, with little or no impact in terms of reducing cardiometabolic outcomes.

Our results showed no effect of cocoa consumption on anthropometric measurements, except for abdominal circumference. However, our analysis was limited, as it included only three RCTs [44,50,58], and cocoa effectiveness was suggested in only one of them [58]. The subgroup analysis by health status (healthy vs. unhealthy individuals with comorbidities) or amount of polyphenol intake found no evidence in favor of cocoa consumption.

As for lipid profile, observational studies have identified high cholesterol and its fractions as modifiable risk factors for coronary artery disease and stroke [67,68,69,70]. A meta-analysis of 61 prospective studies and 55,000 vascular deaths demonstrated that a reduction of 1 mmol/L (38.7 mg/dL) in total cholesterol was associated with a 0.44 (95% CI 0.42 to 0.48 mg/dL) decrease in the risk of death from coronary artery disease in individuals of both sexes aged 40–49 years, a 0.66 decrease risk of death (95% CI 0.65 to 0.68) in those aged 50–69 years, and 0.83 risk of death (95% CI 0.81 to 0.85) in those aged 70–89 years [67]. Another meta-analysis pooled together data from 312,321 participants and found that a reduction in LDL-c levels was associated with a 54.5% decrease (95% CI 48.8 to 59.5%) in the risk of developing coronary heart disease for each one mmol/L (38.7 mg/dL) reduction in LDL-c [69]. Furthermore, Baigent et al. [71] reported a 12% proportional reduction in all-cause mortality for each one mmol/L reduction in LDL-c and, notably, a 19% reduction in coronary ischemic deaths [71]. A mechanistic hypothesis can help explain the observed reduction in total cholesterol and LDL-c that would be attributed to the action of flavonoids present in cocoa [12], especially procyanidins present in the form of monomers, oligomers, or polymers [63,72]. These compounds inhibit the absorption of cholesterol as well as the expression of LDL-c receptors [73,74]. Yet, our study found that cocoa consumption did not change triglyceride and HDL-c levels. The most likely explanation is that triglycerides levels are traditionally sensitive to a decrease in simple sugars and alcohol consumption [75], and these substances are not present in cocoa. Likewise, HDL-c levels are influenced by high consumption of mono and/or polyunsaturated fats (HDL-c) [75], which are also not present in high concentrations in cocoa. It is, thus, reasonable to expect no changes in these markers following polyphenol intake.

Reduced total cholesterol and LDL-C are associated with cardiovascular protection and lower mortality rates. Although our meta-analysis showed modest effects in reducing total cholesterol (−8.35 mg/dL, Figure 3A) and LDL-c (−9.47 mg/dL, Figure 3B), regular dietary intake of cocoa is an interesting strategy for reducing the occurrence of cardiovascular events when considering individual levels of total cholesterol and LDL-c.

Regarding glycemic profile, our systematic review and meta-analysis included 15 studies evaluating fasting glycemia. Our results showed a reduction of −4.91 mg/dL (Figure 4A) in response to cocoa consumption. Also, the subgroup analyses by health status (healthy vs. unhealthy participants with cardiometabolic comorbidities) demonstrated that cocoa consumption led to a reduction in fasting blood glucose of −4.57 mg/dL (95% CI −8.37 to −0.77) only among the participants with an unhealthy status. This effect becomes significant when polyphenol intake is above the median estimated for this outcome. Although cocoa consumption proved to have a beneficial effect on the glycemic profile, HbA1c levels remained unchanged (Figure 3B). However, it is well-known that changes in HbA1c levels become evident after three months. Interestingly, the intervention durations of five studies evaluating HbA1c included in this meta-analysis were as follows: four weeks in the [73,74] study; eight weeks in the [73,74] and [73,74] studies; 12 weeks in the [73,74] study; and 24 weeks in the [73,74] study. A favorable effect on HbA1c was reported in a single RCT with the longest duration, i.e., 24 weeks (Figure 4B). Therefore, other RCTs with longer intervention duration (≥12 weeks) are needed to provide supporting evidence of the effect of cocoa consumption on HbA1c.

Regarding BP levels, our results are in line with the findings reported by Desch, et al. [76] in a meta-analysis that showed a reduction in SBP of −4.5 mmHg and in DBP of −2.5 mmHg following the consumption of dark chocolate and cocoa beverages. In their meta-analysis, Desch et al. [76] examined the effects associated with grape polyphenol consumption and reported a reduction in SBP (−1.48 mmHg) but no effect on DBP [62]. The exact mechanisms for BP reduction associated with the consumption of cocoa products have not yet been fully elucidated. Yet, it has been postulated that it stimulates the formation of endothelial nitric oxide that promotes endothelium-dependent vasodilation, resulting in greater arterial compliance and lower peripheral vascular resistance [77].

Studies on the dietary intake of foods rich in phenolic acids, especially flavonoids such as anthocyanins, have demonstrated vascular protection and increased arterial compliance [61,78]. Our research group has proven that the consumption of anthocyanins, such as açai-jucara fruit (*Euterpe edulis Martius*), effectively protects against arterial stiffness [79], but does not act on endothelium-dependent dilation. Thus, another hypothesis for the reduction of BP levels is that cocoa flavonoids may inhibit the activity of the angiotensin-converting enzyme and promote BP reduction [80,81].

With regard to the amount of polyphenol intake, a double-blind RCT showed reductions of SBP (−3.0 mmHg) and DBP (−1.9 mmHg), even with low amounts of polyphenol intake (30 mg) in individuals with pre-hypertension or stage 1 hypertension [82]. However, our results suggest BP effects only with amounts of cocoa intake above the median calculated from selected RCTs (Figure S20, Supplementary Material File S3). This contrasting finding may be due to the different populations studied. [80,81] evaluated individuals with pre-hypertension or stage 1 hypertension, while our meta-analysis included participants with more diverse characteristics and comorbidities that likely require higher polyphenol amounts to effectively reduce BP levels. In summary, lowering BP even at a modest magnitude has well-documented and important clinical implications; a 4–5 mmHg decrease in SBP can lower cardiovascular risk by 8–20% [62,83]. Our study showed an effect size of −2.52 mmHg for SBP and −1.58 mmHg for DBP. And, this effect may be enhanced at higher amounts of polyphenol intake (SBP −4.66 mmHg; DBP −2.68 mmHg).

We emphasize the need for careful evaluation of dietary intake and/or supplementation of foods rich in polyphenols, including cocoa in pregnant women, especially in the third trimester. Numerous evidence indicate fetal ductus arteriosus constriction due to the anti-inflammatory and antioxidant actions of polyphenols and the inhibition of circulating prostaglandins E2 [84] that may result in fetal complications [85,86]. The literature recommends that pregnant women should limit their intake of foods and supplements rich in polyphenols (≥30 mg/100 g), with a recommended maximum daily intake of 125 mg of polyphenols [87].

Our study has some limitations. The RCTs included in this review evaluated different populations undergoing a variety of interventions in terms of cocoa products consumed (e.g., dry cocoa-extract capsules, >70% cocoa chocolate, cocoa bars or beverages, and cocoa mixed in the meal ingredients); duration (4–24 weeks); and polyphenol and flavonoid concentrations. Therefore, there was relatively high heterogeneity across the studies. However, we used a range of strategies, including subgroup analyses, meta-regressions, overlapping confidence intervals, and sensitivity analyses, to try to explain the source of the heterogeneity found. At the same time, we used a random-effects model to describe the summarized data. Because it takes into account the sample size and the variability of effects across the included studies, it allows for extrapolating the impact of the results to different populations, providing a more comprehensive analysis. Another issue was the studies that evaluated dietary cocoa intake through the consumption of dark chocolate. These products contain different types and amounts of sugar, milk, and chemical ingredients, among others, which may affect some of the outcomes evaluated. Other limitations included studies lacking blinding and/or having inadequate controls (e.g., white chocolate used as a ‘placebo’). Regarding potential biases, we used the RoB2 tool. Yet, this tool does not consider concepts such as the quality of reporting, precision (the extent to which results are free of random errors), or external validity (directness, applicability, or generalization) [64]. However, factors such as journal of publication, year of publication, country of origin, and omission of negative or unpublished data could be a source of location bias, selective (non-) reporting bias, and publication bias in this review. Considering the inclusion of searches in the non-traditional literature, no eligible study showed a high risk of bias by RoB2. Overall, despite these limitations, they do not diminish the validity of the findings in this meta-analysis.

## 5. Conclusions

We conclude that the consumption of cocoa as a dietary supplement in cocoa extract capsules or dark-chocolate products has a protective effect on most cardiometabolic risk markers evaluated in this analysis, including total cholesterol, LDL-c, fasting blood glucose, SBP, and DBP. As for total body weight, BMI, waist or abdominal circumference, triglycerides, and HDL-c, there is no evidence supporting the beneficial effects of cocoa consumption. Thus, we recommend the consumption of cocoa rich in polyphenols as a cardioprotective approach.

Long-term, multicenter well-designed RCTs are needed to confirm or refute our findings. In addition, the positive effects of cocoa should be demonstrated not only in surrogate outcomes but also in clinical trials assessing cardiovascular events in populations in primary and secondary prevention. Nevertheless, considering our results, we suggest that the consumption of polyphenol-rich cocoa could be part of a strategy aimed at promoting cardiovascular health.

## Figures and Tables

**Table 1:** Characteristics of the selected studies.

Reference	Population	n M/F	Groups	Mean Age (Years)	Duration (Weeks)	Outcomes	Intervention (Daily)	Comparator (Control Group)
Ayoobi et al. [35]	Type 2 diabetes mellitus	Total: 44 Sex: 17/27	INT: 21 CG: 23	INT: 50.6 CG: 50.7	8	SBP; DBP; BMI; WC; body weight	84% cocoa beverage (30 g)	Therapeutic lifestyle changes guidelines
Baba et al. [37]	Healthy and mild hypercholesterolemia	Total: 25 Sex: 25 M	INT: 13 CG: 12	INT: 38.0 CG: 38.0	12	SBP; DBP; glucose; BMI; HDL; LDL; cholesterol; triglycerides	26 g cocoa beverage (199 mg of total polyphenols)	Placebo beverage
Baba et al. [36]	Healthy and hypercholesterolemia	Total: 131 Sex: NA	INT 1: 31 INT 2: 32 INT 3: 37 CG: 31	INT 1: 49.0 INT 2: 49.0 INT 3: 49.0 CG: 49.0	4	HDL; LDL; cholesterol; triglycerides	Cocoa beverage INT: 13 g cocoa powder (140.9 mg polyphenols) INT 2: 419.5 g cocoa powder (211.2 mg of polyphenols) INT 3: 26 g cocoa powder (281.5 mg of polyphenols)	Placebo beverage
Balzer et al. [53]	Type 2 diabetes mellitus/drug therapy	Total: 41 Sex: 12/29	INT: 21 CG: 20	INT: 63.1 CG: 64.4	4	Body weight; BMI; WC; glucose; HbA1c. HDL; LDL; cholesterol; triglycerides	Cocoa beverage = mix of cocoa powder + milk powder (963 mg of flavanols)	Cocoa beverage = cocoa extract + milk powder (75 mg of flavanols)
Crews et al. [57]	Healthy elderly	Total: 90 Sex: 38/52	INT: 45 CG: 45	INT: 68.8 CG: 68.7	6	SBP; DBP; BMI; HDL; LDL; cholesterol; triglycerides	One dark-chocolate bar (37.0 g containing 60% cocoa, 11 g natural cocoa, and 397.30 mg of total proanthocyanins/g) and one 8-ounce (237 mL)11 g natural cocoa beverage containing 357.41 mg of total proanthocyanins/g)	Placebo bar and beverage
Davison et al. [40]	Overweight and obese	Total: 49 Sex: 17/32	INT: 25 CG: 24	INT: 45.2 CG: 44.4	12	SBP; DBP; glucose; WC; body weight; BMI; HDL; LDL; cholesterol; triglycerides	High-flavanol cocoa beverage (902 mg of flavanols)	Low-flavanol cocoa beverage (36 mg of flavanols)
Desch et al. [43]	Type 2 diabetes mellitus; hypertension	Total: 91 Sex: 71/20	INT: 43 CG: 48	INT: 62.5 CG: 66.8	24	SBP; DBP	25 g dark-chocolate bar (21 mg of flavanol-epicatechin)	6 g dark-chocolate bar (5 mg of flavanol-epicatechin)
Desideri et al. [38]	Elderly with mild cognitive impairment	Total: 90 Sex: 43/47	INT 1: 30 INT 2: 30 CG: 30	INT 1: 71.3 INT 2: 71.2 CG: 71.0	8	SBP; DBP; glucose; BMI; body weight; HDL; LDL; cholesterol; triglycerides	INT 1: cocoa beverage (520 mg of flavanols) INT 2: cocoa beverage (990 mg of flavanols)	Cocoa beverage (45 mg of flavanols)
Dicks et al. [32]	Type 2 diabetes mellitus; hypertension (stable w/treatment)	Total: 35 Sex: 18/17	INT: 17 CG: 18	INT: 62.8 CG: 65.6	12	SBP; DBP; glucose; HbA1c; BMI; HDL; LDL; cholesterol; triglycerides; WC, body weight	2.5 g cocoa capsules (207 mg of flavanols)	Placebo capsule
Giraldo et al. [50]	Insulin resistance	Total: 75 Sex: 10/65	INT: 37 CG: 38	INT: 49.3 CG: 51.1	8	body weight; BMI; AC; glucose; HDL; LDL; cholesterol; triglycerides	70% dark-chocolate bar (50 g) (430 mg of total polyphenols)	White chocolate with colorant
Ibero-Baraibar et al. [34]	Healthy middle-aged	Total: 47 Sex: 22/25	INT: 24 CG: 23	INT: 57.0 CG: 57.0	4	SBP; DBP, glucose; WC; body weight; HDL; LDL; cholesterol; triglycerides	Meals supplemented with 1.4 g cocoa extract (414.3 mg of flavanols)	Control meals without polyphenols
Jafarirad et al. [52]	Type 2 diabetes mellitus/drug therapy (metformin or glibenclamide)	Total: 44 Sex: 30/14	INT: 21 CG: 23	INT: 52.3 CG: 52.3	8	HbA1c; glucose; HDL; LDL; cholesterol; triglycerides	84% dark chocolate (30 g)	Therapeutic lifestyle changes guidelines
León-Flores et al. [51]	Overweight. serum triglycerides levels of 150–350 mg/dL	Total: 24 Sex: NA	INT: 13 CG: 11	INT: 48.3 CG: 42.0	8	body weight; BMI; WC; Glucose; HDL; LDL; cholesterol; triglycerides	cocoa cookie (25 mg of flavonoids)	Placebo cookie (without cocoa)
Leyva-Soto et al. [58]	Young with 3 cardiovascular risk factors	Total: 84 Sex: 47/37	INT: 42 CG: 42	INT: 23.8 CG: 23.6	24	SBP; DBP; glucose; WC; BMI; HbA1c; HDL; LDL; cholesterol; triglycerides	70% dark-chocolate bar (2 g) (127 mg of total polyphenols/70 mg of flavonoids)	2 g milk chocolate bar (21 mg of flavonoids)
Massee et al. [30]	Healthy young	Total: 40 Sex: 13/27	INT: 20 CG: 20	INT: 24.4 CG: 23.9	4	SBP; DBP	3.05 g cocoa extract (standardized tablet) (250 mg of catechin polyphenol)	Placebo tablet
Mastroiacovo et al. [39]	Healthy elderly	Total: 90 Sex: 37/53	INT 1: 30 INT 2: 30 CG: 30	INT 1: 68.7 INT 2: 70.0 CG: 70.0	8	SBP; DBP; glucose; HbA1c; BMI; HDL; LDL; cholesterol; triglycerides	INT 1: cocoa beverage (520 mg of flavanols) INT 2: cocoa beverage (993 mg of flavanols)	Cocoa beverage (45 mg of flavanols)
Mozaffari-Khosravi et al. [29]	Metabolic syndrome	Total: 94 Sex: 45/49	INT 1: 32 INT 2: 30 CG: 32	INT 1: 49.6 INT 2: 51.7 CG: 52.8	8	SBP; DBP	INT 1: 76% dark-chocolate bar (20 g) (2.46 mg of total polyphenols) INT 2: 76% dark-chocolate bar (40 g) (4.92 mg of total polyphenols)	No placebo
Munguía et al. [44]	Overweight	Total: 15 Sex: 4/11	INT: 5 CG: 10	INT: 41.0 CG: 48.8	4	SBP; DBP, glucose; WC; body weight; BMI; HDL; LDL; cholesterol; triglycerides	Cocoa extract during morning fasting (80 mg of flavonoids)	Placebo powder (without flavonoids)
Munguia et al. [45]	Elderly	Total: 61 Sex: 13/48	INT: 34 CG: 27	INT: 76.1 CG: 75.6	8	body weight, WC. Glucose. HDL; LDL; cholesterol; triglycerides	22 g cocoa beverage (179 mg of flavonoids)	Placebo beverage
Naghdipour-Biregani et al. [54]	Middle-aged/metabolic syndrome	Total: 114 Sex: NA	INT 1: 38 INT 2: 38 CG: 38	INT 1: 49.6 INT 2: 51.8 CG: 52.9	8	HDL; LDL; cholesterol; triglycerides	INT: 76% dark chocolate (20 g) (2.46 mg of total polyphenols) INT 2: 76% dark chocolate (40 g) (4.92 mg of total polyphenols)	No placebo
Neufingerl et al. [49]	Healthy men and postmenopausal women	Total: 69 Sex: 34/35	INT: 35 CG: 34	INT: 55.2 CG: 55.4	4	SBP; DBP; body weight; HDL; LDL; cholesterol; triglycerides	6 g cocoa extract (325 mg of flavonoids)	Placebo beverage
Nickols-Richardson et al. [46]	Overweight and obese premenopausal women	Total: 60 Sex: 60F	INT: 30 CG: 30	INT: 36.0 CG: 36.0	18	SBP; DBP; glucose; WC; BMI; body weight; HDL; LDL; cholesterol; triglycerides	Cocoa beverage + chocolate bar (270 mg of flavonoids)	Placebo snack and beverage (without flavonoids)
Nishiwaki et al. [41]	Healthy young	Total: 32 Sex: 24/8	INT: 16 CG: 16	INT: 20.8 CG: 20.7	4	SBP; DBP; glucose; WC; BMI	72% chocolate bar (20 g) (508 mg of polyphenols)	Without placebo
Ottaviani et al. [31]	Healthy middle-aged	Total: 74 Sex: 33/41	INT: 46 CG: 28	INT: 41.0 CG: 41.0	6	SBP; DBP	1000 mg (2 cocoa-extract capsules) for 2 wks 1500 mg (3 cocoa-extract capsules) for 2 wks 2000 mg (4 cocoa-extract capsules) for 2 wks (500 mg of flavanols/capsule)	Placebo capsule
Parsaeyan et al. [56]	Type 2 diabetes mellitus	Total: 100 Sex: 50/50	INT: 50 CG: 50	INT: 54.0 CG: 54.0	6	HDL; LDL; cholesterol; triglycerides	20 g cocoa extract + 20 g milk powder	Placebo beverage (10 g milk powder)
Pereira et al. [59]	Healthy young	Total: 60 Sex: 20/40	INT: 30 CG: 30	INT: 19.2 CG: 20.7	4	SBP; DBP; BMI	75% chocolate bar (10 g)	No placebo
Pereira et al. [33]	Healthy young	Total: 30 Sex: 4/26	INT: 15 CG: 15	INT: 19.5 CG: 20.4	4	SBP; DBP	90% dark chocolate (20 g) (364 mg of flavanols)	55% chocolate bar (20 g) (252 mg of flavanols)
Polagruto et al. [55]	Hypercholesterolemia	Total: 67 Sex: 20/47	INT: 32 CG: 35	INT: 49.0 CG: 56.0	6	HDL; LDL; cholesterol; triglycerides	Cocoa snack bars (256 mg of flavanols + 3 g sterol esters)	Placebo snack bars (21 mg of flavanols + 0 g sterol esters)
Rodriguez-Mateos et al. [47]	Healthy men	Total: 45 Sex: 45 M	INT 1: 15 INT 2: 15 CG: 15	INT 1: 25.0 INT 2: 23.0 CG: 23.0	4	SBP, DBP; glucose; HDL; LDL; cholesterol; triglycerides	INT 1: cocoa-extract capsules (560 mg of flavanols) INT 2: cocoa-extract capsules (690 mg of flavanols)	Placebo capsule
Rostami et al. [42]	Type 2 diabetes mellitus (stable)/drug therapy; hypertension (stable)	Total: 60 Sex: 24/36	INT: 32 CG: 28	INT: 58.7 CG: 57.2	8	SBP; DBP; glucose; HbA1c; HDL; LDL; cholesterol; triglycerides	83% dark-chocolate bar (25 g) (450 mg of flavonoids)	White chocolate (with colorant)
Sansone et al. [48]	Healthy middle-aged	Total: 105 Sex: 55/50	INT: 55 CG: 50	INT: 45.0 CG: 44.0	4	SBP; DBP, HDL; LDL; cholesterol; triglycerides	cocoa beverage (14 g) (900 mg of flavanols)	Placebo beverage

INT, intervention; CG, control group; SBP, systolic blood pressure; DBP, systolic blood pressure; AC, abdominal circumference; WC, waist circumference; HDL, high-density lipoprotein; LDL, low-density lipoprotein; HbA1c, glycated hemoglobin; BMI, body mass index.

## Data Availability

The databases and script (Rstudio, version 1.3.959, and the R package meta version 3.6.1 for Windows) used in this systematic review for the metanalysis are available on the Mendeley Data repository as open access (https://data.mendeley.com/; doi:10.17632/mwtwh6d8ws.1, accessed on 17 May 2024).

## Supplementary Materials

The following supporting information can be downloaded at https://www.mdpi.com/article/10.3390/nu16121919/s1: Supplementary Material File S1: Search strategy. Supplementary Material File S2: Analyses by health status. Supplementary Material File S3: Analysis by daily amount of polyphenol intake. Supplementary Material File S4: Risk of bias and strength of evidence. Supplementary Material File S5: Sensitivity Analyzes.
