# Supplementary material for: Effects of Cocoa Consumption on Cardiometabolic Risk Markers: Meta-Analysis of Randomized Controlled Trials
Source: Nutrients. 2024 Jun 18;16(12):1919. doi: 10.3390/nu16121919 (PMC11206597; doi:10.3390/nu16121919)
Supplement: Supplementary file 1 [file nutrients-16-01919-s001.zip › Supplementary Material File S1.docx]

**Supplementary Materials S1. Search strategy.**

Effects of Cocoa Consumption on Cardiometabolic Risk
Markers: Meta-Analysis of Randomized Controlled Trials

Tainah O. P. Arisi ^1^, Diego Silveira da Silva ^1^, Elana Stein ^1^, Camila Weschenfelder ^1^,
Patrícia Caetano de Oliveira ^1^, Aline Marcadenti ^1,2,3^, Alexandre Machado Lehnen ^1,^*
and Gustavo Waclawovsky ^1^

^1^ Instituto de Cardiologia do Rio Grande do Sul/Fundação Universitária de Cardiologia,
Porto Alegre 90620-001, RS, Brazil; tainahortiz05@gmail.com (T.O.P.A.); dieguitoef@hotmail.com (D.S.d.S.);
elanast.nutricao@gmail.com (E.S.); camilawesche@gmail.com (C.W.);
fisio.patriciacaetano@gmail.com (P.C.d.O.); marcadenti@yahoo.com.br (A.M.);
gwaclawovsky@gmail.com (G.W.)

^2^ Instituto de Pesquisa Hcor (IP-Hcor), Hcor 04005-909, São Paulo, SP, Brazil

^3^ Faculdade de Saúde Pública, Universidade de São Paulo (FSP-USP), São Paulo 01246-904, SP, Brazil

***** Correspondence: amlehnen@gmail.com; Tel.: +55-(51)-3230-3600 (ext. 3636/3757)

| **Table S1. Search terms for searches in Medline, EMBASE, Web of Science, Cochrane, LILACS and SciELO** |
| --- |
| **MEDLINE (PubMed)** |
| (((Chocolate OR Chocolates OR Cocoa OR Cocoa Powders OR Cocoa Powders OR Powder, Cocoa OR Powders, Cocoa OR Cacao OR Cocoa Plant OR Plant, Cocoa OR Theobroma OR Theobroma cacao)) AND ((Blood Pressure OR Pressure, Blood OR Diastolic Pressure OR Pressure, Diastolic OR Systolic Pressure OR Pressure, Systolic OR Pressures, Systolic OR Arterial Pressures OR Pressure, Arterial OR Pressures, Arterial OR Arterial Tension OR Arterial Tensions OR Tension, Arterial OR Tensions, Arterial OR Blood Pressure, Arterial OR Arterial Blood Pressure OR Arterial Blood Pressures OR Blood Pressures, Arterial OR Pressure, Arterial Blood OR Pressures, Arterial Blood OR Mean Arterial Pressures OR Pressure, Mean Arterial OR Pressures, Mean Arterial OR Ambulatory Blood Pressure Monitoring OR Monitoring, Ambulatory Blood Pressure OR Blood Pressure Monitoring Ambulatory Blood Pressure Monitoring OR Body Mass OR Body Weights OR Weight, Body OR Weights, Body OR Index, Body Mass OR Quetelet Index OR Index, Quetelet OR Quetelet's Index OR Quetelets Index OR Cholesterol OR HDL Cholesterol OR High Density Lipoprotein Cholesterol OR alpha-Lipoprotein Cholesterol OR Cholesterol, alpha-Lipoprotein OR alpha Lipoprotein Cholesterol OR HDL Lipoproteins OR High-Density Lipoprotein OR Lipoprotein, High-Density OR High-Density Lipoproteins OR High Density Lipoproteins OR Lipoproteins, High-Density OR High Density Lipoprotein OR Density Lipoprotein, High OR Lipoprotein, High Density OR Cholesterol, alpha-Lipoprotein OR High Density Lipoprotein Cholesterol OR HDL2 Cholesterol OR HDL3 Cholesterol OR alpha-Lipoprotein Cholesterol OR Low Density Lipoprotein Cholesterol OR beta-Lipoprotein Cholesterol OR Cholesterol, beta-Lipoprotein OR beta Lipoprotein Cholesterol OR LDL Cholesterol OR Cholesteryl Linoleate, LDL OR LDL Cholesteryl Linoleate OR VLDL Cholesterol OR Pre-beta-Lipoprotein Cholesterol OR Cholesterol, Pre-beta-Lipoprotein OR Pre beta Lipoprotein Cholesterol OR Very Low Density Lipoprotein Cholesterol OR Pre beta lipoprotein Cholesterol OR Cholesterol, Pre beta lipoprotein OR Blood Sugar OR Sugar, Blood OR Glucose, Blood OR Hemoglobin, Glycated OR Glycohemoglobin OR Glycohemoglobins OR Glycated Hemoglobins OR Hemoglobins, Glycated OR Hemoglobin, Glycosylated OR Glycosylated Hemoglobin OR Glycated Hemoglobin A1c OR Hemoglobin A1c, Glycated OR Glycosylated Hemoglobin A1c OR Hemoglobin A1c, Glycosylated Hb A1a-2 OR Hemoglobin, Glycated A1a-2 OR A1a-2 Hemoglobin, Glycated OR Glycated A1a-2 Hemoglobin OR Hemoglobin, Glycated A1a 2 OR Glycated Hemoglobin A OR Hemoglobin A, Glycated OR Hb A1a+b OR Hb A1c OR HbA1 OR Glycosylated Hemoglobin A OR Hemoglobin A, Glycosylated OR Hb A1 OR Glycohemoglobin A OR Hemoglobin, Glycosylated A1a-1 OR A1a-1 Hemoglobin, Glycosylated OR Glycosylated A1a-1 Hemoglobin OR Hemoglobin, Glycosylated A1a 1 OR Hb A1a-1 OR Hemoglobin, Glycated A1b OR A1b Hemoglobin, Glycated OR Glycated A1b Hemoglobin OR Hb A1b OR Hemoglobin, Glycosylated A1b OR A1b Hemoglobin, Glycosylated OR Glycosylated A1b Hemoglobin))) AND (Randomized controlled trial[pt] OR controlled clinical trial[pt] OR randomized controlled trials[mh] OR random allocation[mh] OR dORble-blind method[mh] OR single-blind method[mh] OR clinical trial[pt] OR clinical trials[mh] OR "clinical trial"[tw] OR singl*[tw] OR dORbl*[tw] OR trebl*[tw] OR tripl*[tw] OR random*[tw] OR cross-over studies[mh] OR control*[tw] OR volunteer*[tw]) |
| **EMBASE** |
| chocolate'/exp OR 'cacao'/exp AND 'blood pressure' OR 'arterial pressure'/exp OR 'blood pressure monitoring'/exp AND 'cholesterol'/exp OR 'low density lipoprotein'/exp OR 'high density lipoprotein'/exp OR 'cholesterol blood level'/exp OR 'very low-density lipoprotein'/exp AND 'hemoglobin A1c'/exp AND 'body mass'/exp AND 'randomized controlled trial'/exp |
| **COCHRANE** |
| ((Chocolates OR Cocoa Powder OR Powder, Cocoa OR Cocoa Powders OR Powders, Cocoa) AND (Glucose, Blood OR Blood Sugar OR Sugar, Blood OR Hemoglobin A1c, Glycated OR Hemoglobin A1c, Glycosylated OR Glycosylated Hemoglobin A1c OR Glycated Hemoglobin A1c OR Hemoglobin, Glycosylated A1b OR Glycosylated A1b Hemoglobin OR A1b Hemoglobin, Glycated OR A1b Hemoglobin, Glycosylated OR Hb A1bOR Glycated A1b Hemoglobin OR Hemoglobin, Glycated A1b OR A1a-1 Hemoglobin, Glycosylated OR Hemoglobin, Glycosylated A1a-1 OR Hemoglobin, Glycosylated A1a 1 OR Glycosylated A1a-1 Hemoglobin OR Hb A1a-1 OR Hemoglobin, Glycated A1a 2 OR A1a-2 Hemoglobin, Glycated OR Hemoglobin, Glycated A1a-2 OR Hb A1a-2 OR Glycated A1a-2 Hemoglobin OR Glycosylated Hemoglobin OR Hemoglobin, Glycosylated OR Glycated Hemoglobins OR Hemoglobins, Glycated OR Glycated Hemoglobin OR Hemoglobin, Glycated OR Hb A1c OR Hb A1 OR Glycohemoglobin A OR Hemoglobin A(1) OR Hemoglobin A, Glycated OR Glycosylated Hemoglobin A OR Hemoglobin A, Glycosylated OR Hb A1a+b OR HbA1 OR Blood Pressure OR Pressure, Blood OR Diastolic Pressure OR Pressure, Diastolic OR Pulse Pressure OR Pressure, Pulse OR Systolic Pressure OR Pressures, Systolic OR Pressure, Systolic OR Blood Pressure, Arterial OR Tensions, Arterial OR Arterial Blood Pressures OR Blood Pressures, Arterial OR Pressures, Arterial Blood OR Arterial Blood Pressure OR Pressure, Arterial Blood OR Arterial Pressures OR Arterial Tensions OR Pressure, Arterial OR Pressures, Arterial OR Arterial Tension OR Tension, Arterial OR Pressure, Mean Arterial OR Arterial Pressures, Mean OR Arterial Pressure, Mean OR Pressures, Mean Arterial OR Mean Arterial Pressure OR Mean Arterial Pressures OR Blood Pressure Monitoring, Home OR Home Blood Pressure Monitoring OR Ambulatory Blood Pressure Monitoring OR Monitoring, Ambulatory Blood Pressure OR Self Blood Pressure Monitoring OR Blood Pressure Monitoring, Self OR Body Weight OR Body Weights OR Weight, Body OR Weights, Body OR Body Weight Change OR Change, Body Weight OR Changes, Body Weight OR Body Weight Maintenance OR Maintenances, Body Weight OR Maintenance, Body Weight OR Body Weight Maintenances OR Ideal Body Weight OR Ideal Body Weight Formula OR Normal Body Weight OR Body Weight, Normal OR Body Weights, Normal OR Body Weights, Ideal OR Ideal Body Weights OR Body Weight, Ideal OR Normal Body Weights OR Ideal Body Weight Chart OR Body-Weight Trajectories OR Trajectories, Body-Weight OR Body Weight Trajectory OR Trajectory, Body-Weight OR Body Mass Index OR Quetelet's Index OR Quetelet Index OR Index, Quetelet OR Quetelets Index OR Index, Body Mass OR Blood Cholesterol OR High Density Lipoprotein Cholesterol OR Cholesterol, alpha-Lipoprotein OR alpha Lipoprotein Cholesterol OR alpha-Lipoprotein Cholesterol OR HDL(2) Cholesterol OR HDL(3) Cholesterol OR HDL Cholesterol OR HDL2 Cholesterol OR Cholesterol, HDL2 OR Cholesterol, HDL3 OR HDL3 Cholesterol OR Cholesterol, LDL OR Low Density Lipoprotein Cholesterol OR beta Lipoprotein Cholesterol OR beta-Lipoprotein Cholesterol OR LDL Cholesterol OR Cholesterol, beta-Lipoprotein OR LDL Cholesteryl Linoleate OR Cholesteryl Linoleate OR Very Low Density Lipoprotein Cholesterol OR Cholesterol, Pre-beta-Lipoprotein OR Pre-beta-Lipoprotein Cholesterol OR Pre beta Lipoprotein Cholesterol OR Cholesterol, Pre beta lipoprotein OR Pre beta lipoprotein Cholesterol OR VLDL Cholesterol)) |
| **LILACS** |
| English: (Cocoa OR Cocoa Plant OR Plant, Cocoa OR Theobroma OR Theobroma cacao OR Chocolates OR Chocolate OR Cocoa Powder OR Cocoa Powders OR Powder, Cocoa OR Powders, Cocoa) AND (Blood Pressure OR systolic OR diastolic OR BMI OR Body Weights OR Body Mass Index OR Blood Glucose OR Glycated Hemoglobin OR Cholesterol OR HDL OR LDL OR VLDL))  Portuguese: (Cacau OR Cacau Planta OR Planta, Cacau OR Theobroma OR Theobroma cacao OR Chocolates OR Chocolate OR Cacau em Pó OR Cacau em Pó OR Pó, Cacau OR Pós, Cacau) and (pressão arterial OR sistólica OR diastólica OR IMC OR peso corporal OR índice de massa corporal OR glicose OR hemoglobina glicada OR colesterol OR HDL OR LDL OR VLDL)  Spanish: ((Cacao OR Planta de cacao OR Planta, Cacao OR Theobroma OR Theobroma cacao OR Chocolates OR Chocolate OR Cacao en polvo OR Cacao en polvo OR Polvo, Cacao OR Polvos, Cacao) and (Presión arterial OR sistólica OR diastólica OR IMC OR peso corporal OR Pesos corporales OR índice de massa corporal OR Glucosa OR hemoglobina glucosilada OR colesterol OR HDL OR LDL OR VLDL)) |
| **WEB OF SCIENCE** |
| (Chocolate OR Chocolates OR Cocoa OR Cocoa Powders OR Cocoa Powders OR Powder, Cocoa OR Powders, Cocoa OR Cacao OR Cocoa Plant OR Theobroma) AND (Blood Pressure OR Body Mass OR Body Weights OR Cholesterol OR Cholesterol Total OR HDL Cholesterol OR High Density Lipoprotein Cholesterol OR LDL Cholesterol OR Blood Sugar OR Glucose, Blood OR Glycated Hemoglobin A1c) AND (Randomized controlled trial OR controlled clinical trial OR clinical trial OR random OR cross-over studies) |
| **SciELO (Advanced Searches)** |
| (Cocoa OR Chocolates OR Chocolate) AND (Blood Glucose) OR (Glycated Hemoglobin) OR (Blood Pressure) OR (Body Weights) OR (Body Mass Index) OR (Cholesterol) |
