# Supplementary material for: Effects of Cocoa Consumption on Cardiometabolic Risk Markers: Meta-Analysis of Randomized Controlled Trials
Source: Nutrients. 2024 Jun 18;16(12):1919. doi: 10.3390/nu16121919 (PMC11206597; doi:10.3390/nu16121919)
Supplement: Supplementary file 1 [file nutrients-16-01919-s001.zip › Supplementary Material File S2.docx]

**Supplementary Materials S2. Analyses by health status.**

Effects of Cocoa Consumption on Cardiometabolic Risk
Markers: Meta-Analysis of Randomized Controlled Trials

Tainah O. P. Arisi ^1^, Diego Silveira da Silva ^1^, Elana Stein ^1^, Camila Weschenfelder ^1^,
Patrícia Caetano de Oliveira ^1^, Aline Marcadenti ^1,2,3^, Alexandre Machado Lehnen ^1,^* and Gustavo Waclawovsky ^1^

^1^ Instituto de Cardiologia do Rio Grande do Sul/Fundação Universitária de Cardiologia,
Porto Alegre 90620-001, RS, Brazil; tainahortiz05@gmail.com (T.O.P.A.); dieguitoef@hotmail.com (D.S.d.S.);
elanast.nutricao@gmail.com (E.S.); camilawesche@gmail.com (C.W.);
fisio.patriciacaetano@gmail.com (P.C.d.O.); marcadenti@yahoo.com.br (A.M.);
gwaclawovsky@gmail.com (G.W.)

^2^ Instituto de Pesquisa Hcor (IP-Hcor), Hcor 04005-909, São Paulo, SP, Brazil

^3^ Faculdade de Saúde Pública, Universidade de São Paulo (FSP-USP), São Paulo 01246-904, SP, Brazil

***** Correspondence: amlehnen@gmail.com; Tel.: +55-(51)-3230-3600 (ext. 3636/3757)


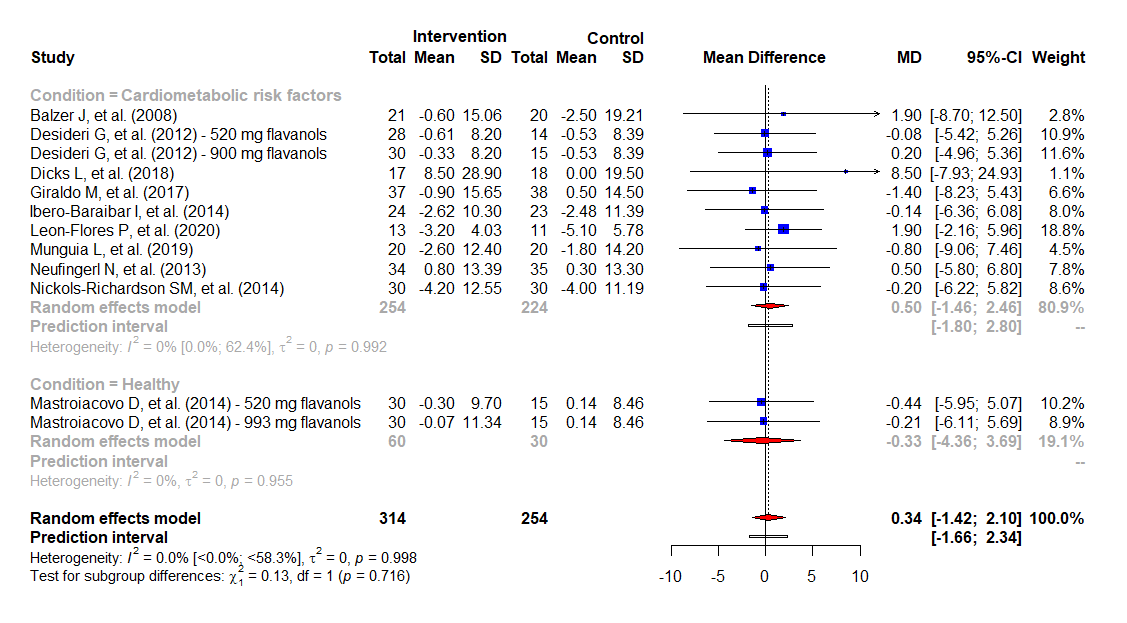


**Figure S1**. Effects of cocoa consumption vrs control on Body Weight by Health Condition. Cardiometabolic risk factors: RCTs that used people with established cardiometabolic risk factors as arterial hypertension, and/or type 2 diabetes mellitus, and/or dyslipidemia, and/or overweight/obesity, and/or myocardial infarction/stroke. Healthy: RCTs that involved people with no cormibidities established..


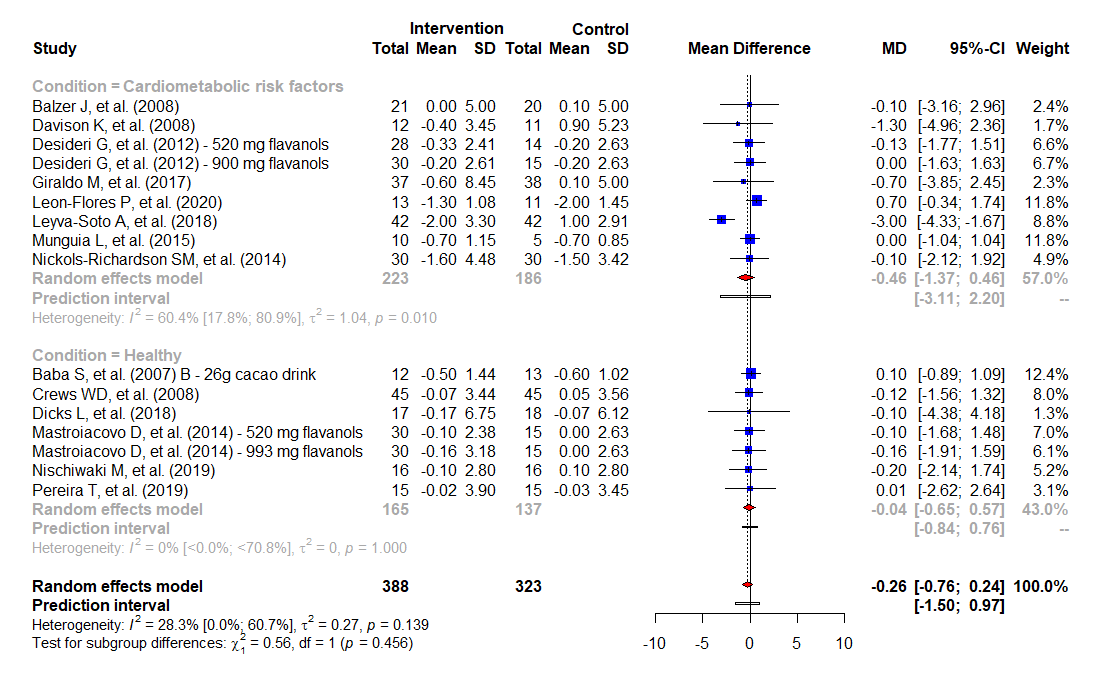


**Figure S2**. Effects of cocoa consumption vrs control on Body Mass Index by Health Condition. Cardiometabolic risk factors: RCTs that used people with established cardiometabolic risk factors as arterial hypertension, and/or type 2 diabetes mellitus, and/or dyslipidemia, and/or overweight/obesity, and/or myocardial infarction/stroke. Healthy: RCTs that involved people with no cormibidities established.


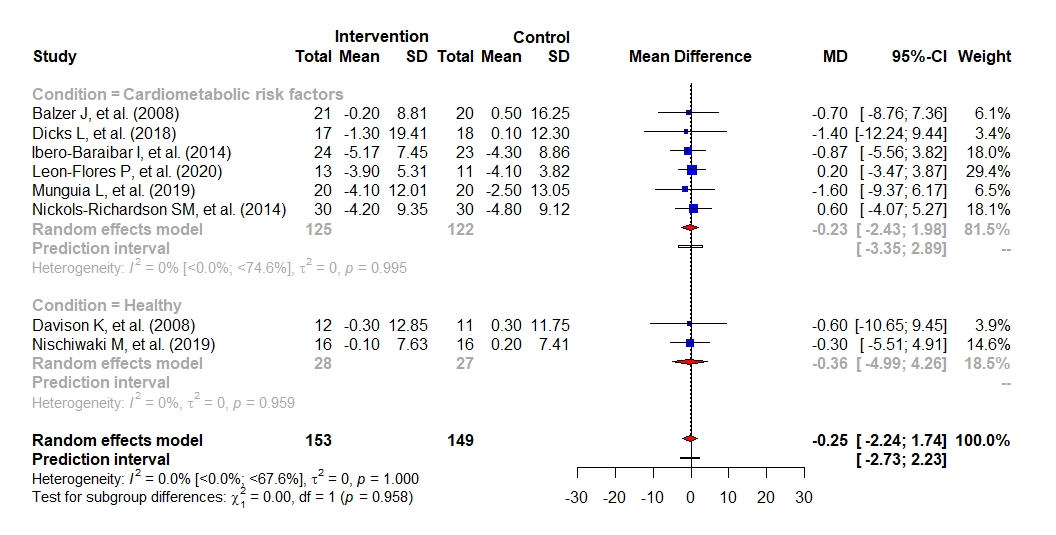


**Figure S3**. Effects of cocoa consumption vrs control on Waist Circumference by Health Condition. Cardiometabolic risk factors: RCTs that used people with established cardiometabolic risk factors as arterial hypertension, and/or type 2 diabetes mellitus, and/or dyslipidemia, and/or overweight/obesity, and/or myocardial infarction/stroke. Healthy: RCTs that involved people with no cormibidities established.


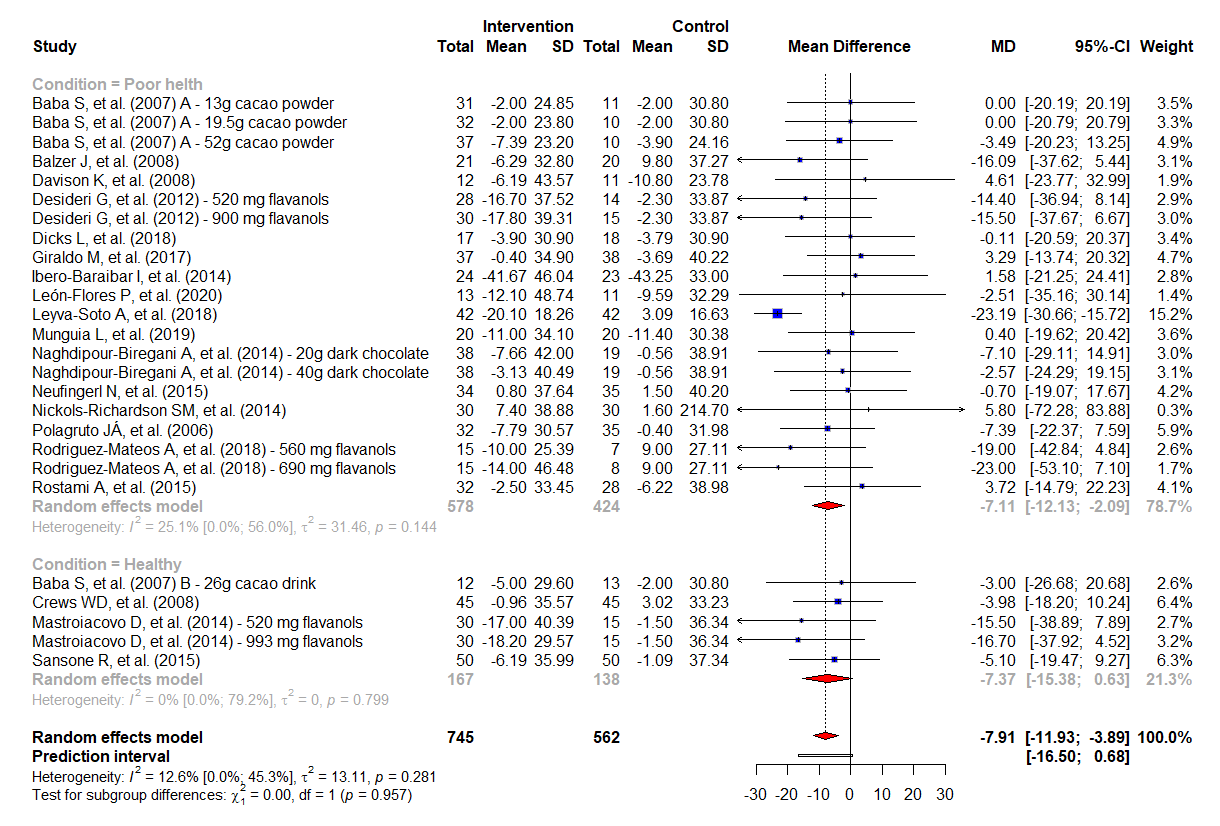


**Figure S4.** Effects of cocoa consumption vrs control on Total Cholesterol by Health Condition. Cardiometabolic risk factors: RCTs that used people with established cardiometabolic risk factors as arterial hypertension, and/or type 2 diabetes mellitus, and/or dyslipidemia, and/or overweight/obesity, and/or myocardial infarction/stroke. Healthy: RCTs that involved people with no cormibidities established.


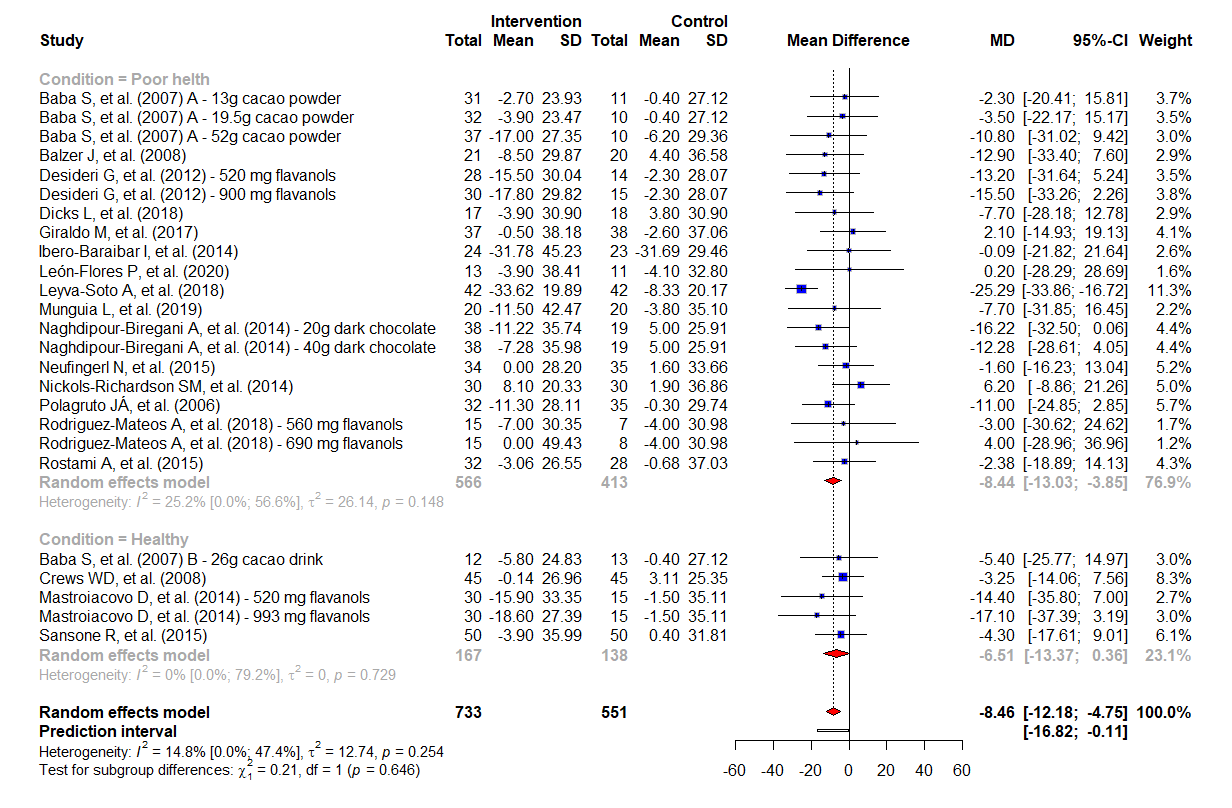


**Figure S5**. Effects of cocoa consumption vrs control on Low Density Lipoprotein Cholesterol by Health Condition. Cardiometabolic risk factors: RCTs that used people with established cardiometabolic risk factors as arterial hypertension, and/or type 2 diabetes mellitus, and/or dyslipidemia, and/or overweight/obesity, and/or myocardial infarction/stroke. Healthy: RCTs that involved people with no cormibidities established.


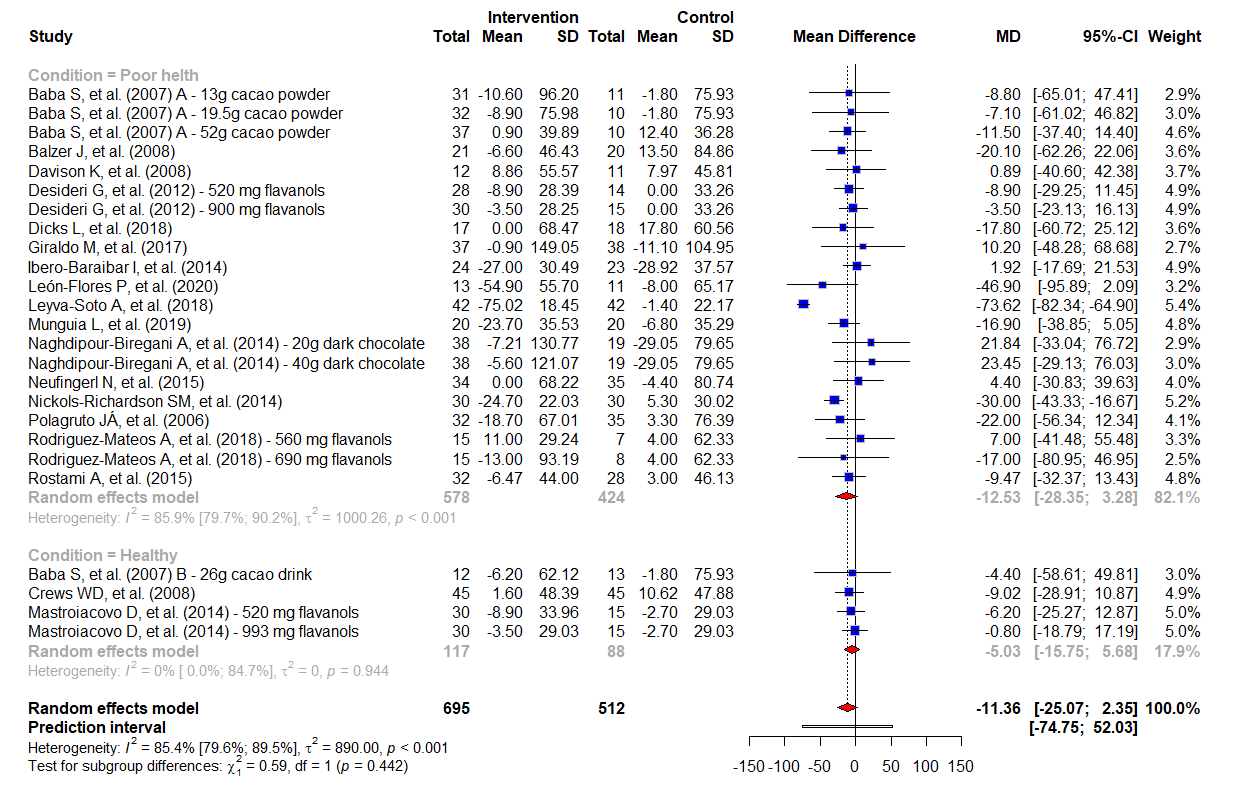


**Figure S6.** Effects of cocoa consumption vrs control on Triglycerides by Health Condition. Cardiometabolic risk factors: RCTs that used people with established cardiometabolic risk factors as arterial hypertension, and/or type 2 diabetes mellitus, and/or dyslipidemia, and/or overweight/obesity, and/or myocardial infarction/stroke. Healthy: RCTs that involved people with no cormibidities established.


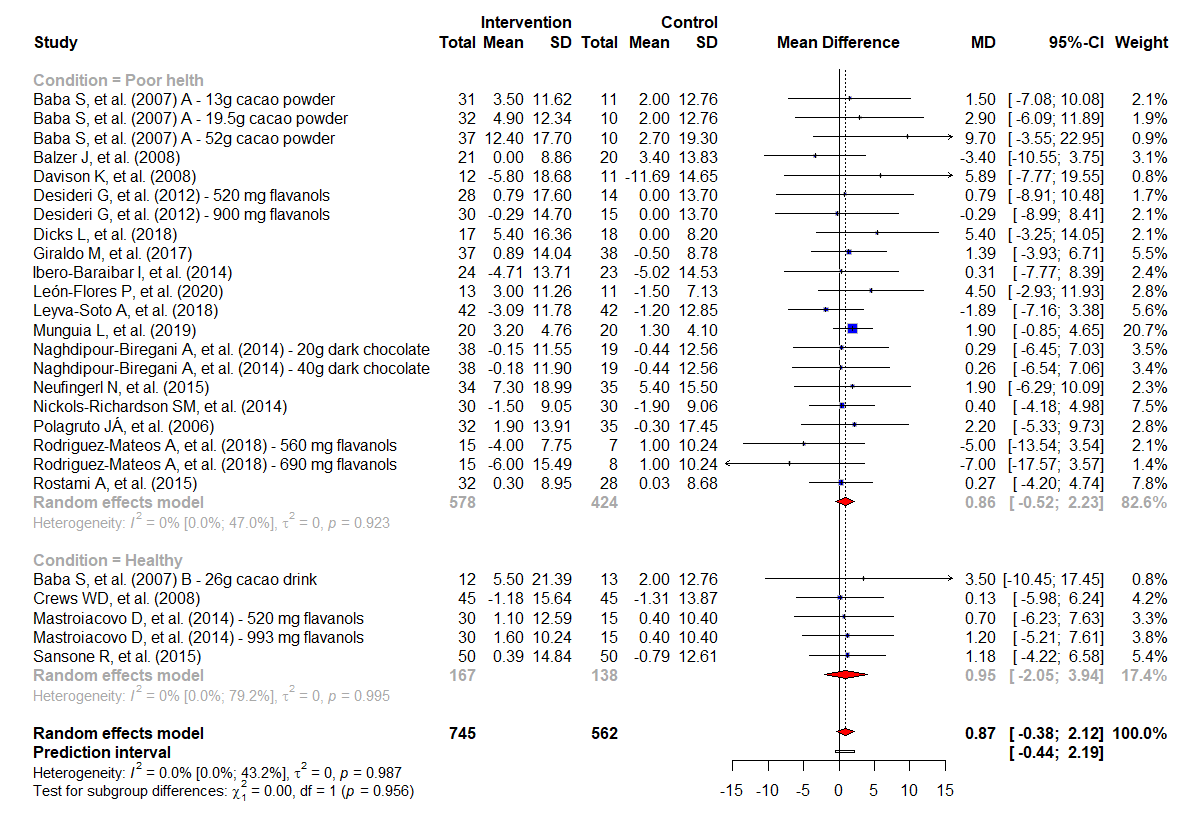


**Figure S7**. Effects of cocoa consumption vrs control on High Density Lipoprotein Cholesterol by Health Condition. Cardiometabolic risk factors: RCTs that used people with established cardiometabolic risk factors as arterial hypertension, and/or type 2 diabetes mellitus, and/or dyslipidemia, and/or overweight/obesity, and/or myocardial infarction/stroke. Healthy: RCTs that involved people with no cormibidities established.


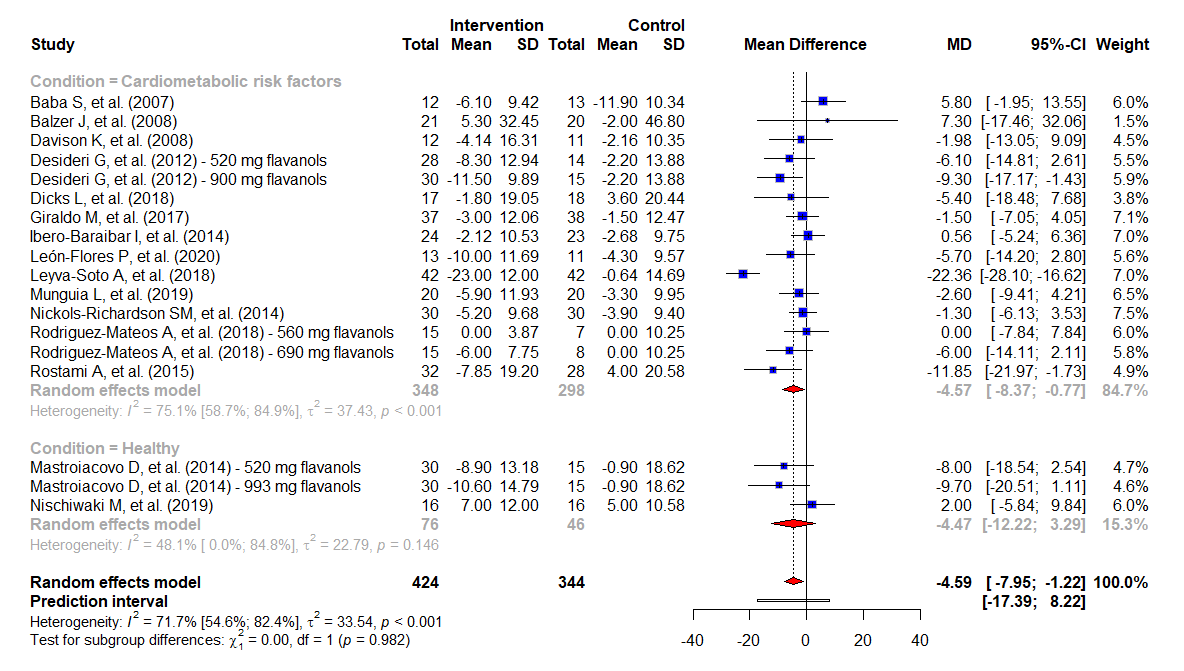


**Figure S8.** Effects of cocoa consumption vrs control on Fasting Blood Glucose by Health Condition. Cardiometabolic risk factors: RCTs that used people with established cardiometabolic risk factors as arterial hypertension, and/or type 2 diabetes mellitus, and/or dyslipidemia, and/or overweight/obesity, and/or myocardial infarction/stroke. Healthy: RCTs that involved people with no cormibidities established.


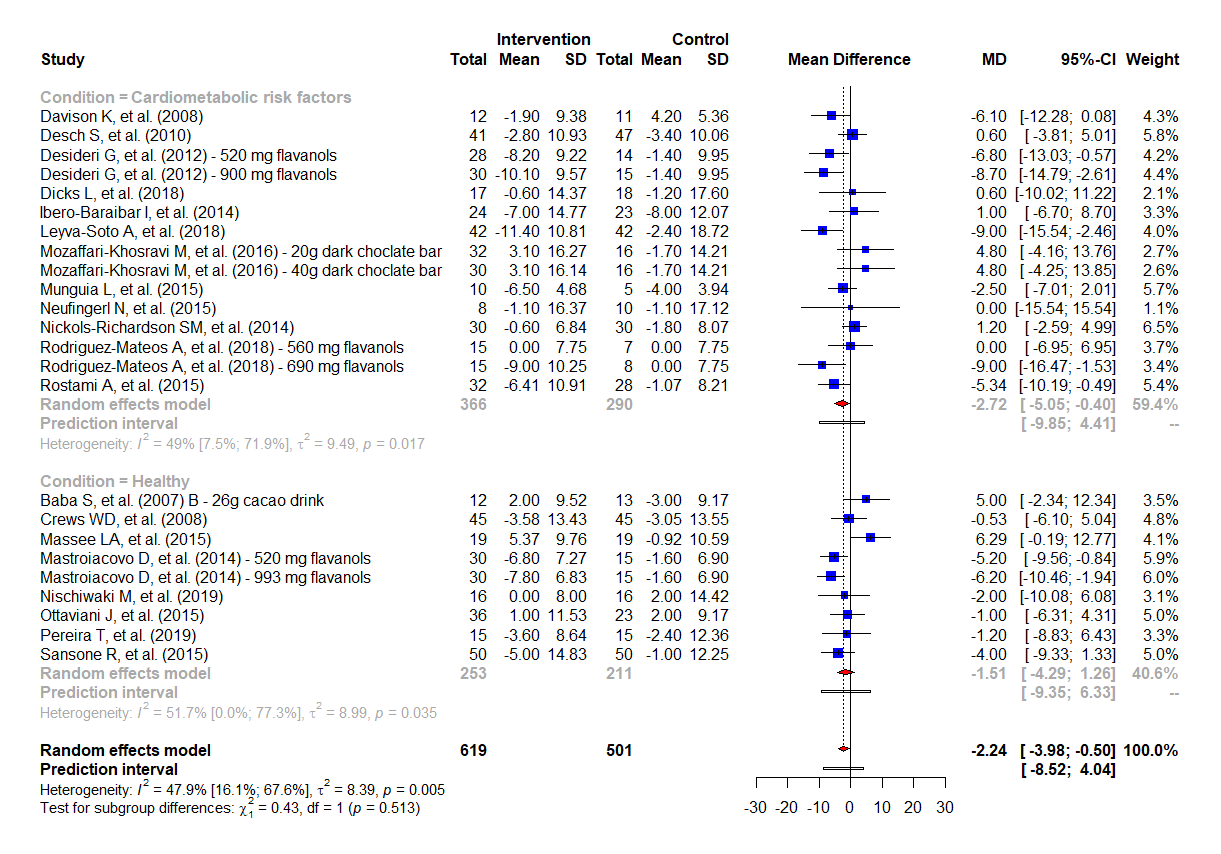


**Figure S9**. Effects of cocoa consumption vrs control on Systolic Blood Pressure by Health Condition. Cardiometabolic risk factors: RCTs that used people with established cardiometabolic risk factors as arterial hypertension, and/or type 2 diabetes mellitus, and/or dyslipidemia, and/or overweight/obesity, and/or myocardial infarction/stroke. Healthy: RCTs that involved people with no cormibidities established.


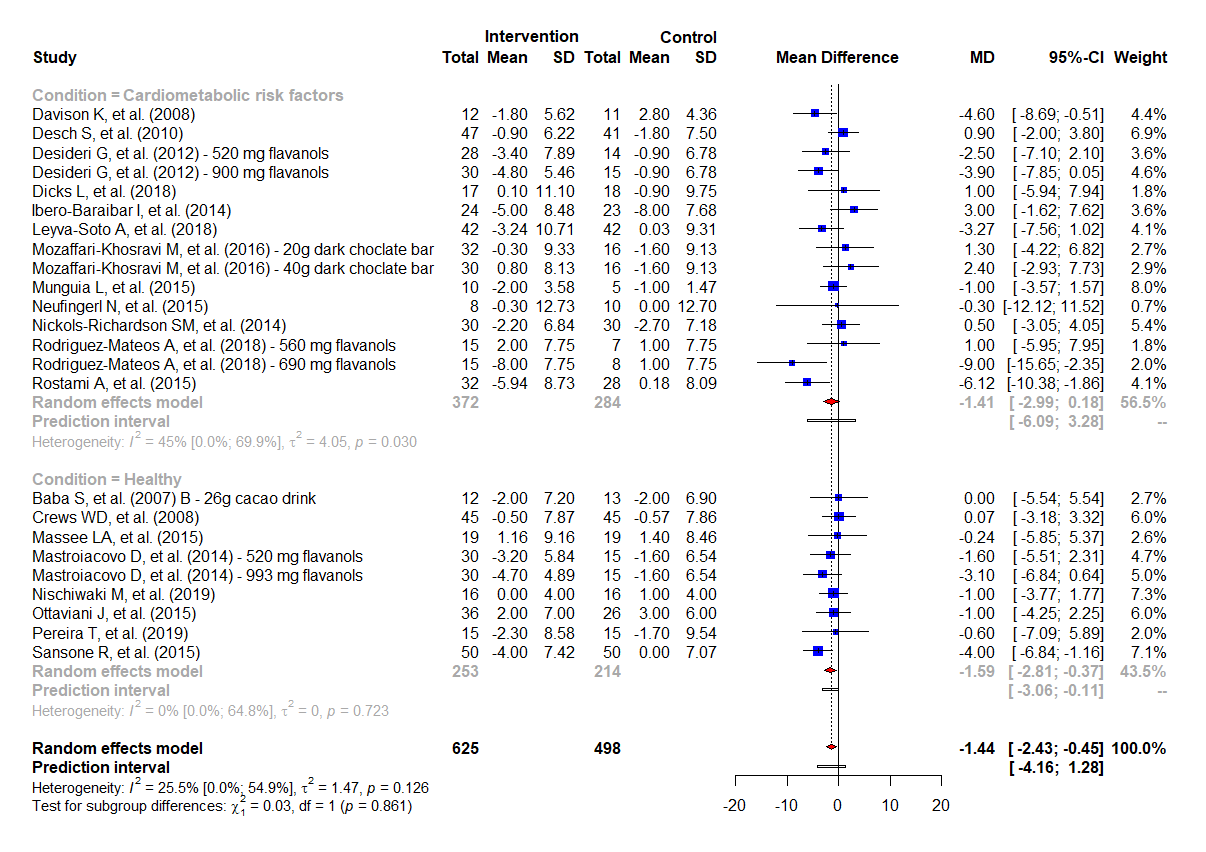


**Figure S10**. Effects of cocoa consumption vrs control on Diastolic Blood Pressure by Health Condition. Cardiometabolic risk factors: RCTs that used people with established cardiometabolic risk factors as arterial hypertension, and/or type 2 diabetes mellitus, and/or dyslipidemia, and/or overweight/obesity, and/or myocardial infarction/stroke. Healthy: RCTs that involved people with no cormibidities established.
