# Supplementary material for: Effects of Cocoa Consumption on Cardiometabolic Risk Markers: Meta-Analysis of Randomized Controlled Trials
Source: Nutrients. 2024 Jun 18;16(12):1919. doi: 10.3390/nu16121919 (PMC11206597; doi:10.3390/nu16121919)
Supplement: Supplementary file 1 [file nutrients-16-01919-s001.zip › Supplementary Material File S4.docx]

**Supplementary Materials S4. Risk of bias and strength of evidence.**

Effects of Cocoa Consumption on Cardiometabolic Risk
Markers: Meta-Analysis of Randomized Controlled Trials

Tainah O. P. Arisi ^1^, Diego Silveira da Silva ^1^, Elana Stein ^1^, Camila Weschenfelder ^1^,
Patrícia Caetano de Oliveira ^1^, Aline Marcadenti ^1,2,3^, Alexandre Machado Lehnen ^1,^* and Gustavo Waclawovsky ^1^

^1^ Instituto de Cardiologia do Rio Grande do Sul/Fundação Universitária de Cardiologia,
Porto Alegre 90620-001, RS, Brazil; tainahortiz05@gmail.com (T.O.P.A.);
dieguitoef@hotmail.com (D.S.d.S.); elanast.nutricao@gmail.com (E.S.); camilawesche@gmail.com (C.W.);
fisio.patriciacaetano@gmail.com (P.C.d.O.); marcadenti@yahoo.com.br (A.M.);
gwaclawovsky@gmail.com (G.W.)

^2^ Instituto de Pesquisa Hcor (IP-Hcor), Hcor 04005-909, São Paulo, SP, Brazil

^3^ Faculdade de Saúde Pública, Universidade de São Paulo (FSP-USP), São Paulo 01246-904, SP, Brazil

***** Correspondence: amlehnen@gmail.com; Tel.: +55-(51)-3230-3600 (ext. 3636/3757)


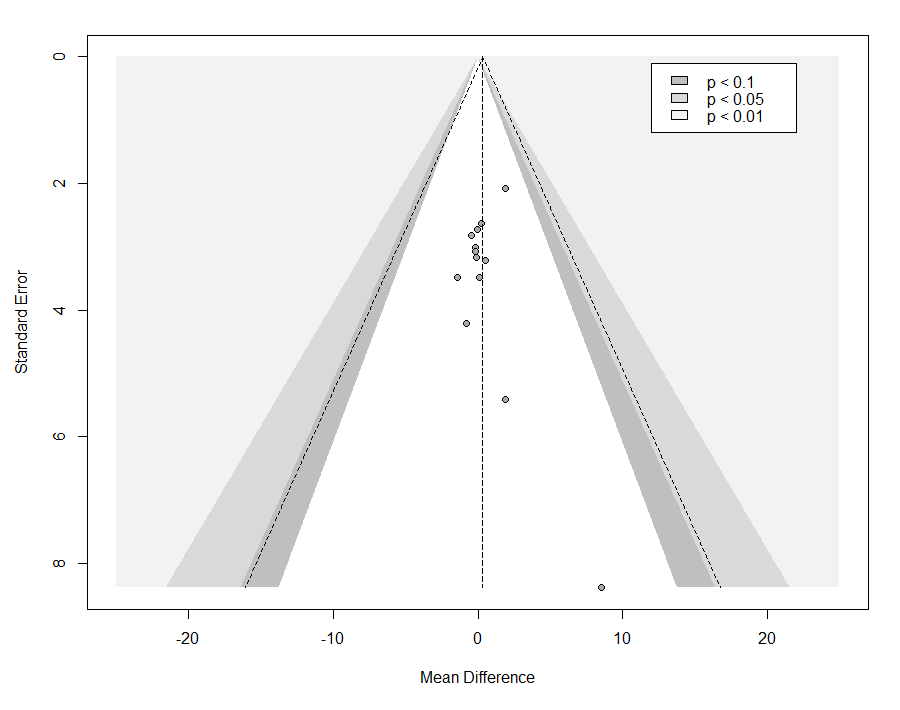


**Figure S21.** Funnel plot for risk of publication bias relative to Body Weight. Test result: t = 0.68, df = 11, p-value = 0.508, bias estimate: 0.298 (SE = 0.435).


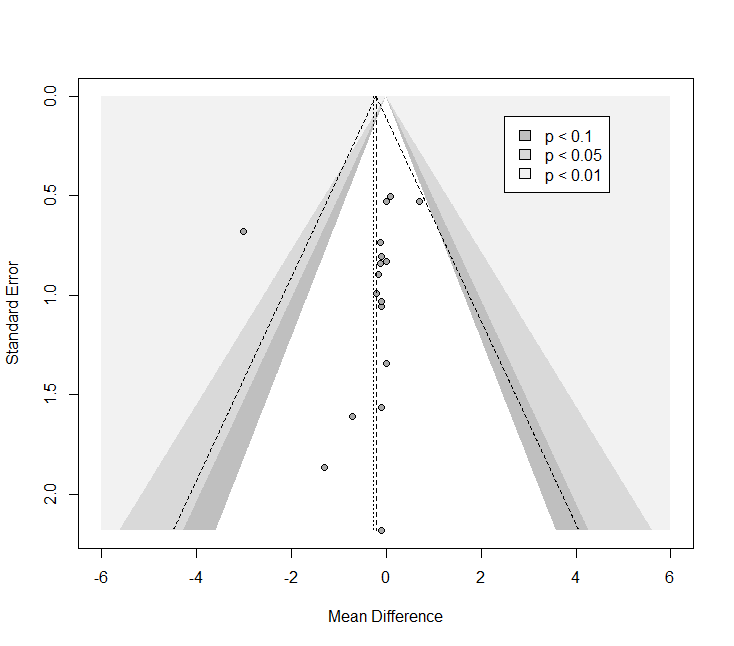


**Figure S22.** Funnel plot for risk of publication bias relative to Body Mass Index. Test result: t = -0.56, df = 15, p-value = 0.582, bias estimate: -0.424 (SE = 0.754).


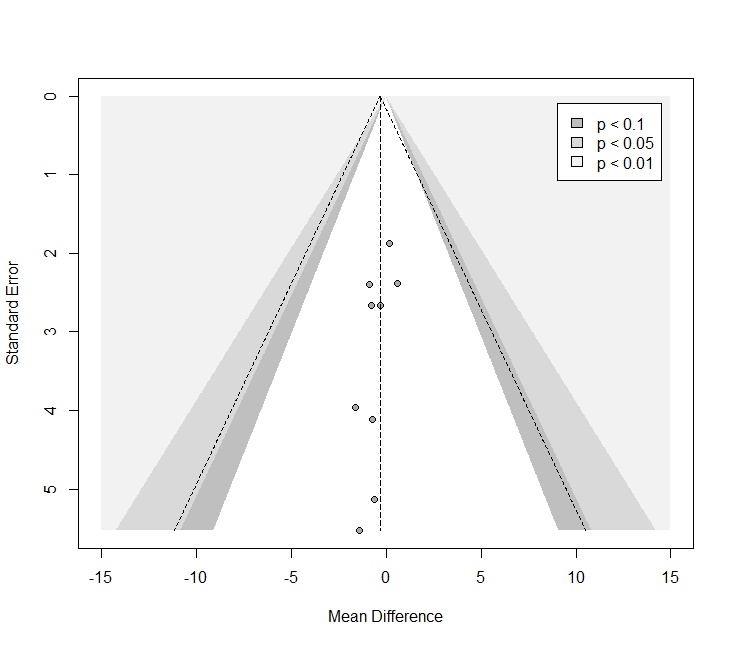


**Figure S23.** Funnel plot for risk of publication bias relative to Waist Circumference. Test result: t = 2.04, df = 7, p-value = 0.081, bias estimate: -0.431 (SE = 0.211).


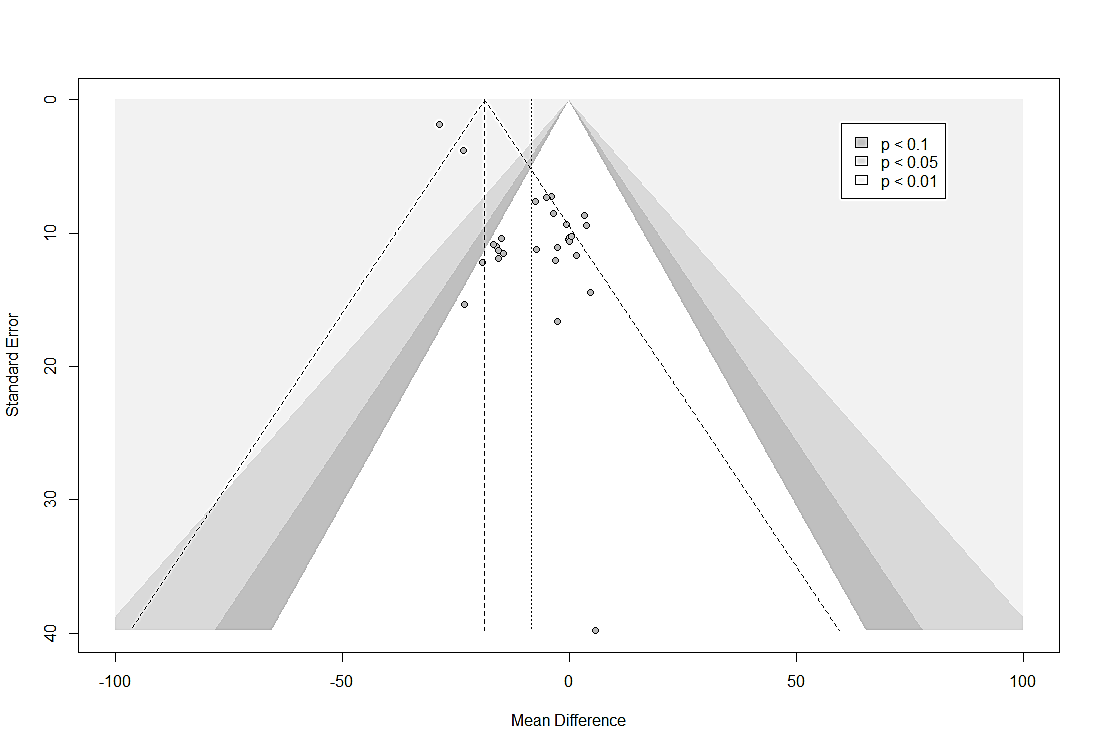


**Figure S24**. Funnel plot for risk of publication bias relative to Total Cholesterol. Test result: t = 7.56, df = 26, p-value < 0.001, bias estimate: 2.323 (SE = 0.307).


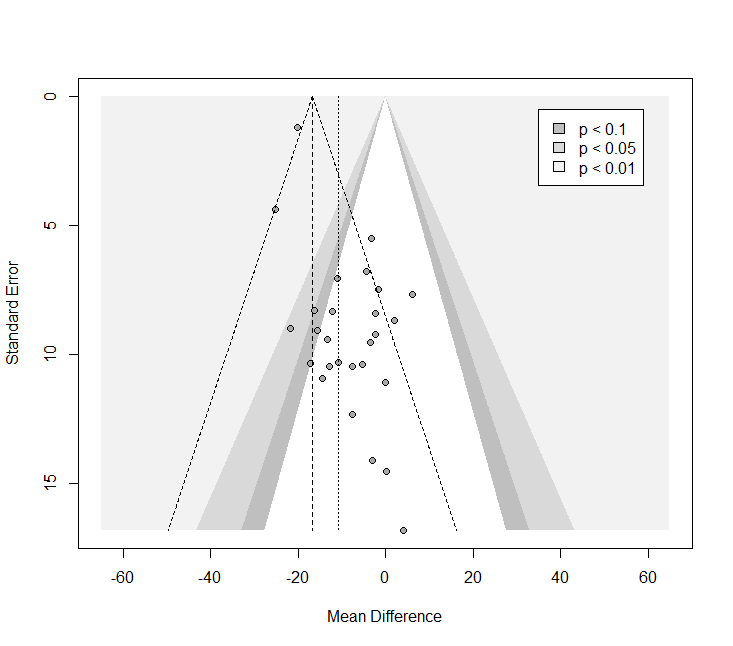


**Figure S25.** Funnel plot for risk of publication bias relative to Low Density Liporotein Cholesterol. Test result: t = 3.05, df = 26, p-value = 0.005, bias estimate: 1.218 (SE = 0.400).


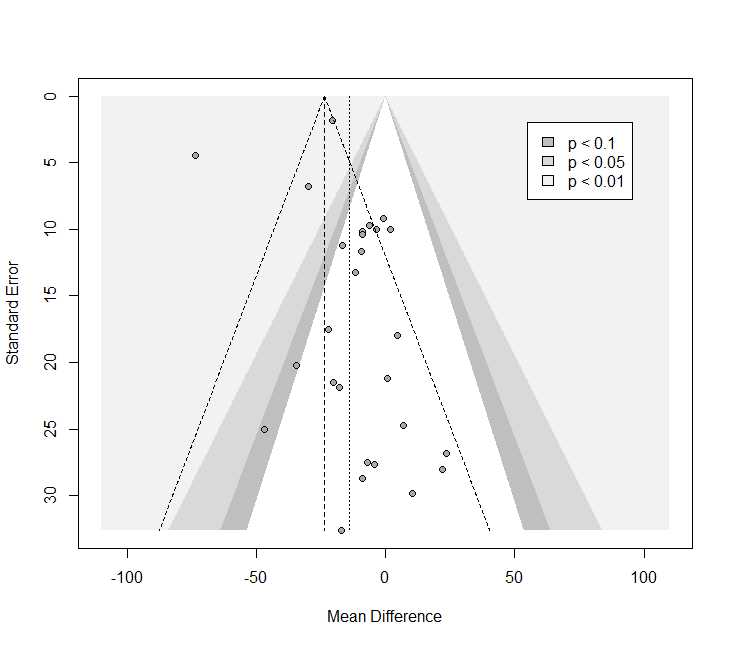


**Figure S26.** Funnel plot for risk of publication bias relative to Triglycerides. Test result: t = 1.37, df = 25, p-value = 0.183, bias estimate: 0.892 (SE = 0.651).


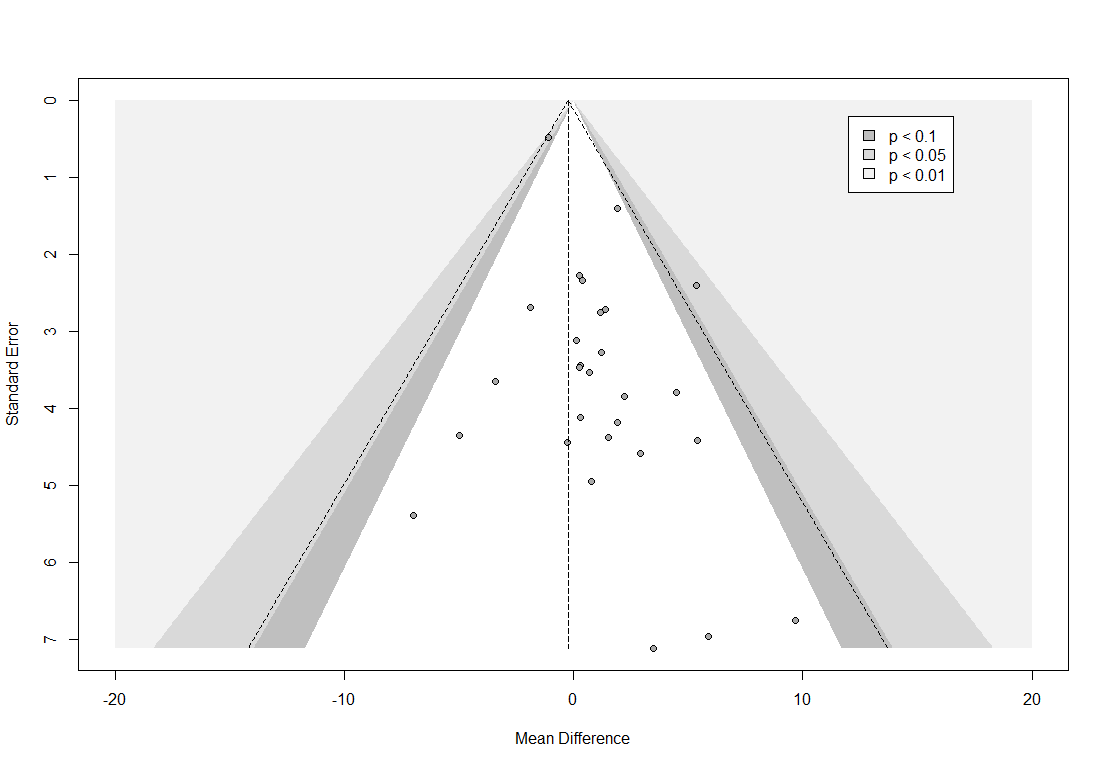


**Figure S27**. Funnel plot for risk of publication bias relative to High Density Liporotein Cholesterol. Test result: t = 2.90, df = 26, p-value = 0.008, bias estimate: 0.640 (SE = 0.221).


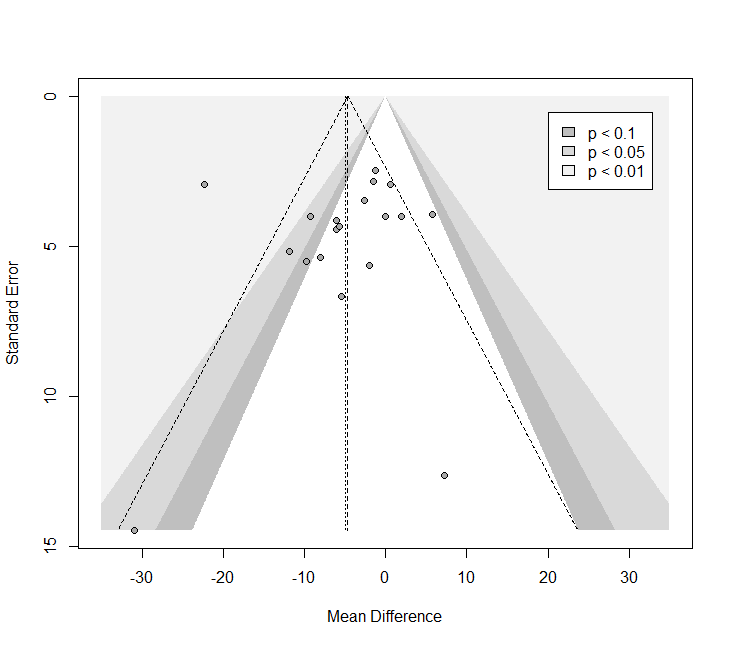


**Figure S28.** Funnel plot for risk of publication bias relative to Fasting Boold Glucose. Test result: t = 0.42, df = 17, p-value = 0.680, bias estimate: -0.541 (SE = 1.291).


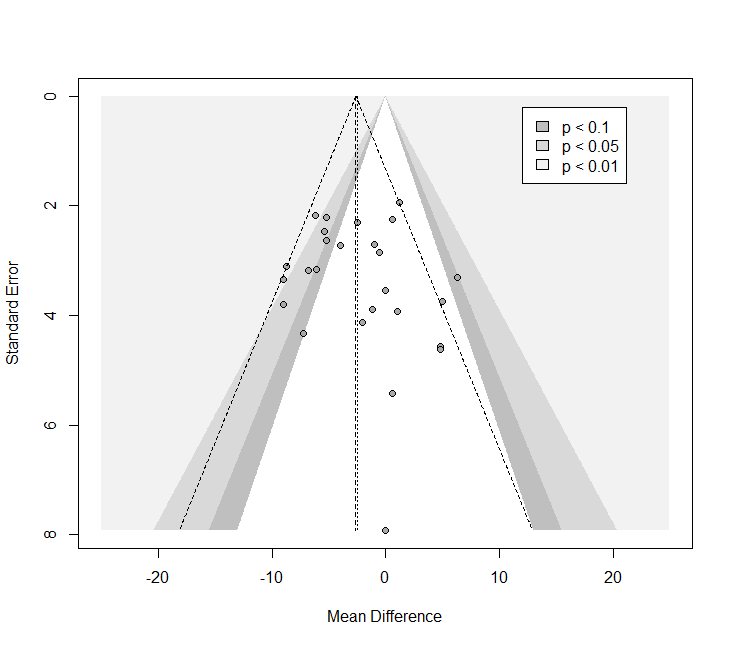


**Figure S29**. Funnel plot for risk of publication bias relative to Systolic Blood Pressure. Test result: t = 0.70, df = 24, p-value = 0.488, bias estimate: 0.675 (SE = 0.957).


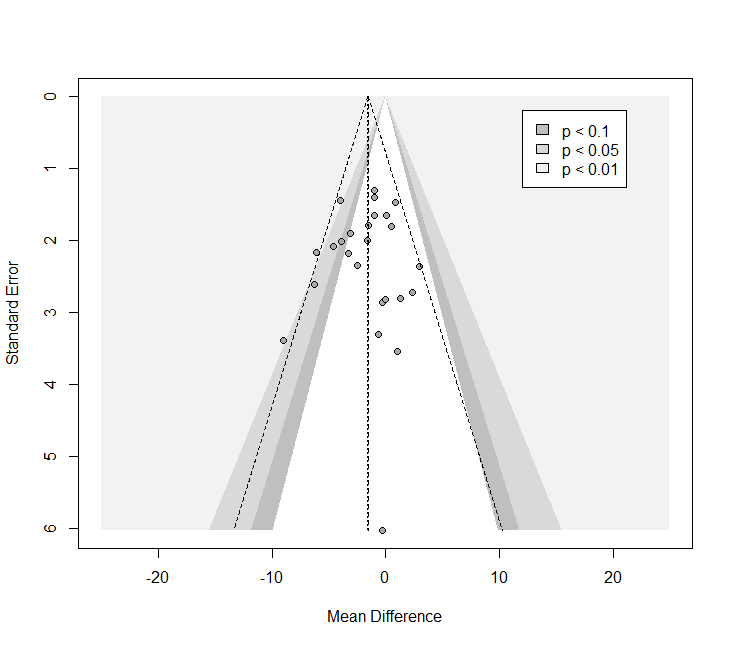


**Figure S30.** Funnel plot for risk of publication bias relative to Diastolic Blood Pressure. Test result: t = -0.11, df = 24, p-value = 0.910, vias estimate: -0.088 (SE = 0.769).


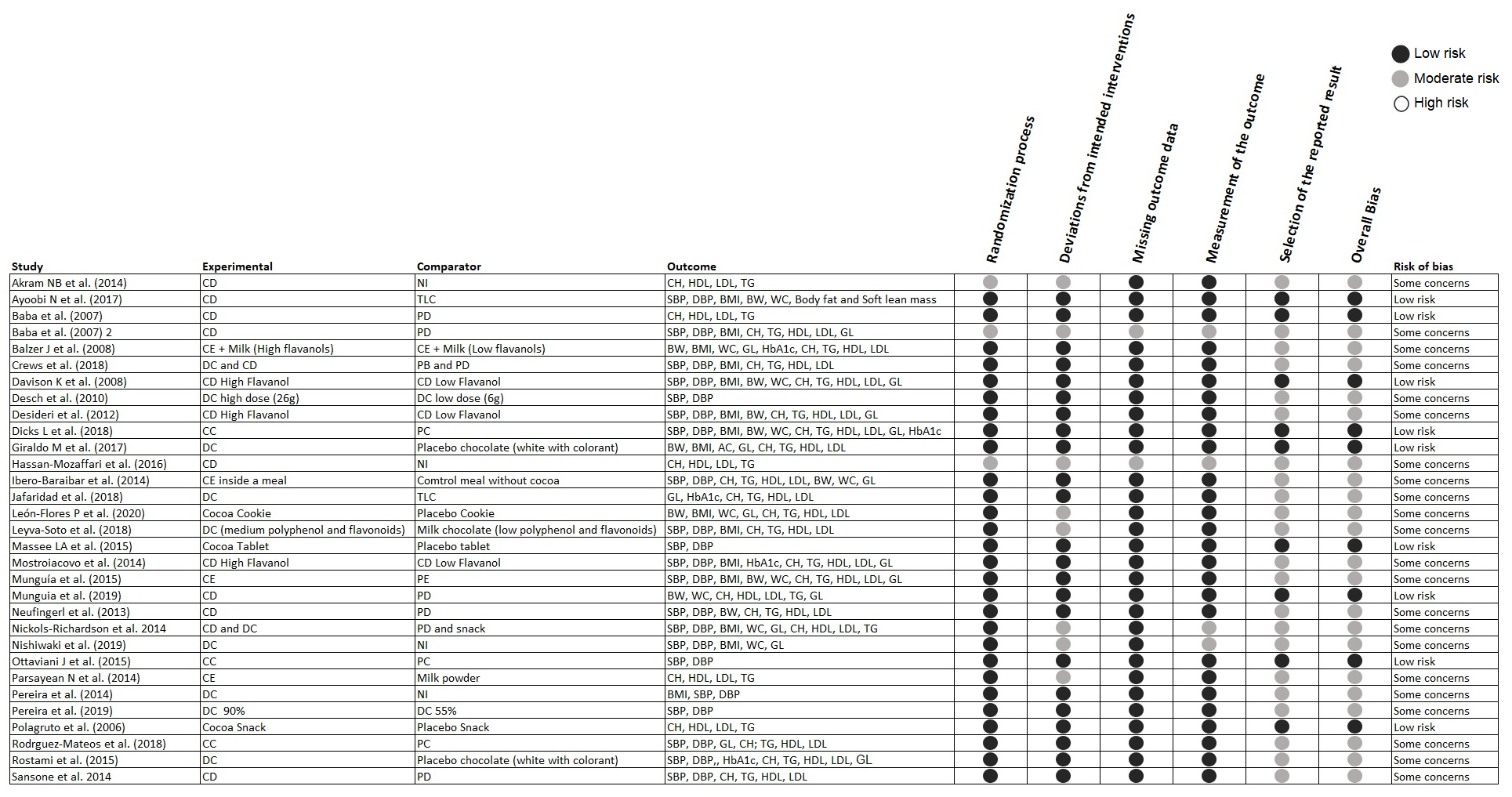


**Figure S31**. Risk of structured bias from RoB2. SBP: systolic blood pressure, DBP: diastolic blood pressure, GL: glucose, CH: cholesterol, TG: triglycerides, HDL: high-density lipoprotein, LDL low-density lipoprotein, BMI: body mass index, BW: body weight, CC: cocoa capsules, DC: dark chocolate bar, CD: cocoa drink, CE: cocoa extract, PB: placebo bar, PC: placebo capsule, PD: placebo drink, PE: placebo extract, NI: no intervention, WC: white chocolate, TLC: therapeutic lifestyle changes guidelines.

| **Table S2. The strength of evidence by GRADE regarding cocoa consumption on anthropometric parameters** | | | | | | |  |
| --- | --- | --- | --- | --- | --- | --- | --- |
| **Patient or Population:**  adults **Setting:** outpatient **Intervention:** cocoa consumption **Comparison:** control group without intervention or placebo | | | | | | |  |
| **Outcomes** | | **Potential absolute effects*** (95% CI)  **Risk with cocoa consumption** | | **Relative effect (95% CI)** | **№ of participants (studies)** | **Certainty of the evidence (GRADE)** | **Comments** |
| Total body mass | |  | **MD** **0.33 kg higher** (1.38 lower to 2.03 higher) | - | 612 (11 RCTs) | ⨁⨁⨁◯ MODERATE ^a, b^ | Cocoa consumption likely results in a slight or no increase in total body mass. Even with a low level of heterogeneity in the studies included, more studies are still needed to confirm whether the results found are reliable. |
| Body Mass Index | |  | **MD** **0.25 kg lower**  (0.72 lower to 0.22 higher) | - | 755 (15 RCTs) | ⨁⨁⨁◯ MODERATE ^a, b^ | Cocoa consumption probably results in a reduction in body mass index, but more studies are needed on the subject to confirm the results found and infer good confidence in the outcome results. |
| Waist circumference | |  | **MD** **0.32 cm lower**  (2.18 lower to 1.54 higher) | - | 346 (9 RCTs) | ⨁⨁⨁◯ MODERATE ^a, b^ | Cocoa consumption probably results in a reduction in waist circumference, but not significant among the groups studied. More studies on the topic are still needed. |
| Abdominal circumference | |  | **MD** **3.93 cm lower**  (7.66 lower to 0.20 lower) | - | 174 (3 RCTs) | ⨁⨁⨁◯ MODERATE ^a, b^ | Cocoa consumption likely results in a reduction in abdominal circumference. However, more studies are still needed to confirm the results found and thus infer confidence in high certainty in the data for this outcome. |
| * The risk in the intervention group (and its 95% confidence interval) is based on the assumed risk in the comparison group and the absolute effect of the intervention (and its 95% CI).  CI: Confidence interval; MD: mean difference  GRADE Working Group grades of evidence  High certainty: We are very confident that the true effect lies close to that of the estimate of the effect  Moderate certainty: We are moderately confident in the effect estimate: The true effect is likely to be close to the estimate of the effect, but there is a possibility that it is substantially different  Low certainty: Our confidence in the effect estimate is limited: The true effect may be substantially different from the estimate of the effect  Very low certainty: We have very little confidence in the effect estimate: The true effect is likely to be substantially different from the estimate of effect  a. Due to moderate heterogeneity for the effects of grouped means, due to the size and characteristic differences between sample participants. However, we followed recommendations^1^ and used the random model to try to explain potential effect modifiers, excluding discrepant data through the lack of overlapping 95% CI and using subgroup analysis and/or meta-regression. Furthermore, we added in our analysis the range of uncertainty of the effect, described by the IP 95%.  b. We can consider a moderate imprecision of the estimated effect due to the null line being crossed by the CI and the range of CIs that we can consider relevant for some outcomes.  ^1^ Higgins JPT, Cochrane Collaboration. Cochrane handbook for systematic reviews of interventions. Second edition. ed. Wiley-Blackwell; 2019:xxviii, 694 pages. | | | | | | |  |

| **Table S3. The strength of evidence by GRADE regarding cocoa consumption on lipid profile** | | | | | | |  |
| --- | --- | --- | --- | --- | --- | --- | --- |
| **Patient or Population:**  adults **Setting:** outpatient **Intervention:** cocoa consumption **Comparison:** control group without intervention or placebo | | | | | | |  |
| **Outcomes** | | **Potential absolute effects*** (95% CI)  **Risk with cocoa consumption** | | **Relative effect (95% CI)** | **№ of participants (studies)** | **Certainty of the evidence (GRADE)** | **Comments** |
| Total cholesterol | |  | **MD** **8.37mg/dL lower**  (14.03 lower to 2.70 lower) | - | 1451 (22 RCTs) | ⨁⨁⨁◯ MODERATE ^a, b^ | Cocoa consumption probably results in a reduction in total cholesterol. Even with low heterogeneity, more studies are still needed to confirm the results found and thus infer confidence in a high certainty in the data for this outcome. |
| Low-density lipoprotein | |  | **MD** **0.67mg/dL lower**  (15.49 lower to 5.85 lower) | - | 1451 (22 RCTs) | ⨁⨁⨁◯ MODERATE ^a, b^ | Cocoa consumption probably results in a reduction in LDL levels, but further studies on the topic are recommended to improve the level of confidence. |
| Triglycerides | |  | **MD** **13.37mg/dL lower**  (24.01 lower to 2.72 lower) | - | 1351 (21 RCTs) | ⨁⨁⨁◯ MODERATE ^a, b^ | Cocoa consumption probably results in a reduction in total cholesterol, more studies can confirm the results found and thus infer confidence in a high certainty in the data for this outcome. |
| High-density lipoprotein | |  | **MD** **0.28mg/dL lower** (1.02 lower to 0.46 higher) | - | 1451 (22 RCTs) | ⨁⨁⨁◯ MODERATE ^a, b^ | Cocoa consumption probably results in a slight or no reduction in HDL levels, more study on the subject is needed to investigate the outcome and improve your level of confidence |
| * The risk in the intervention group (and its 95% confidence interval) is based on the assumed risk in the comparison group and the absolute effect of the intervention (and its 95% CI).  CI: Confidence interval; MD: mean difference  GRADE Working Group grades of evidence  High certainty: We are very confident that the true effect lies close to that of the estimate of the effect  Moderate certainty: We are moderately confident in the effect estimate: The true effect is likely to be close to the estimate of the effect, but there is a possibility that it is substantially different  Low certainty: Our confidence in the effect estimate is limited: The true effect may be substantially different from the estimate of the effect  Very low certainty: We have very little confidence in the effect estimate: The true effect is likely to be substantially different from the estimate of effect  a. Due to moderate heterogeneity for the effects of grouped means, due to the size and characteristic differences between sample participants. However, we followed recommendations^1^ and used the random model to try to explain potential effect modifiers, excluding discrepant data through the lack of overlapping 95% CI and using subgroup analysis and/or meta-regression. Furthermore, we added in our analysis the range of uncertainty of the effect, described by the IP 95%.  b. We can consider a moderate imprecision of the estimated effect due to the null line being crossed by the CI and the range of CIs that we can consider relevant for some outcomes.  ^1^ Higgins JPT, Cochrane Collaboration. Cochrane handbook for systematic reviews of interventions. Second edition. ed. Wiley-Blackwell; 2019:xxviii, 694 pages. | | | | | | |  |

| **Table S4. The strength of evidence by GRADE regarding cocoa consumption on glycemic profile** | | | | | | |  |
| --- | --- | --- | --- | --- | --- | --- | --- |
| **Patient or Population:**  adults **Setting:** outpatient **Intervention:** cocoa consumption **Comparison:** control group without intervention or placebo | | | | | | |  |
| **Outcomes** | | **Potential absolute effects*** (95% CI)  **Risk with cocoa consumption** | | **Relative effect (95% CI)** | **№ of participants (studies)** | **Certainty of the evidence (GRADE)** | **Comments** |
| Fasting blood glucose | |  | **MD** **4.91mg/dL lower**  (8.29 lower to 1.52 lower) | - | 752 (16 RCTs) | ⨁⨁⨁◯ MODERATE ^a, b^ | Cocoa consumption probably results in a reduction in blood glucose, but further studies are still needed to confirm the results found and thus infer confidence in a high certainty in the data for this outcome. |
| Glycated hemoglobin (HbA1c) | |  | **MD** **0.36mg/dL lower**  (0.86 lower to 0.14 higher) | - | 264 (5 RCTs) | ⨁⨁⨁◯ MODERATE ^a, b^ | Cocoa consumption probably results in a reduction in glycated hemoglobin levels, but further studies on the topic are recommended to improve the level of confidence. |
| * The risk in the intervention group (and its 95% confidence interval) is based on the assumed risk in the comparison group and the absolute effect of the intervention (and its 95% CI).  CI: Confidence interval; MD: mean difference  GRADE Working Group grades of evidence  High certainty: We are very confident that the true effect lies close to that of the estimate of the effect  Moderate certainty: We are moderately confident in the effect estimate: The true effect is likely to be close to the estimate of the effect, but there is a possibility that it is substantially different  Low certainty: Our confidence in the effect estimate is limited: The true effect may be substantially different from the estimate of the effect  Very low certainty: We have very little confidence in the effect estimate: The true effect is likely to be substantially different from the estimate of effect  a. Due to moderate heterogeneity for the effects of grouped means, due to the size and characteristic differences between sample participants. However, we followed recommendations^1^ and used the random model to try to explain potential effect modifiers, excluding discrepant data through the lack of overlapping 95% CI and using subgroup analysis and/or meta-regression. Furthermore, we added in our analysis the range of uncertainty of the effect, described by the IP 95%.  b. We can consider a moderate imprecision of the estimated effect due to the null line being crossed by the CI and the range of CIs that we can consider relevant for some outcomes.  ^1^ Higgins JPT, Cochrane Collaboration. Cochrane handbook for systematic reviews of interventions. Second edition. ed. Wiley-Blackwell; 2019:xxviii, 694 pages. | | | | | | |  |

| **Table S5. The strength of evidence by GRADE regarding cocoa consumption on blood pressure** | | | | | | |  |
| --- | --- | --- | --- | --- | --- | --- | --- |
| **Patient or Population:** adults **Setting:** outpatient **Intervention:** cocoa consumption **Comparison:** control group without intervention or placebo | | | | | | |  |
| **Outcomes** | | **Potential absolute effects*** (95% CI)  **Risk with cocoa consumption** | | **Relative effect (95% CI)** | **№ of participants (studies)** | **Certainty of the evidence (GRADE)** | **Comments** |
| Systolic blood pressure (SBP) | |  | **MD** **2.52 mmHg lower**  (4.17 lower to 0.88 lower) | - | 1224 (22 RCTs) | ⨁⨁⨁◯ MODERATE ^a, b^ | Cocoa consumption probably results in a reduction in systolic blood pressure, however, more studies on the subject are recommended to confirm the results found and thus infer confidence in a high certainty in the data for this outcome. |
| Diastolic blood pressure (DBP) | |  | **MD** **1.58 mmHg lower**  (2.54 lower to 0.62 lower) | - | 1224 (22 RCTs) | ⨁⨁⨁◯ MODERATE ^a, b^ | Cocoa consumption probably results in a reduction in diastolic blood pressure, however, more studies on the subject are recommended to confirm the results found and thus infer confidence in a high certainty in the data for this outcome. |
| * The risk in the intervention group (and its 95% confidence interval) is based on the assumed risk in the comparison group and the absolute effect of the intervention (and its 95% CI).  CI: Confidence interval; MD: mean difference  GRADE Working Group grades of evidence  High certainty: We are very confident that the true effect lies close to that of the estimate of the effect  Moderate certainty: We are moderately confident in the effect estimate: The true effect is likely to be close to the estimate of the effect, but there is a possibility that it is substantially different  Low certainty: Our confidence in the effect estimate is limited: The true effect may be substantially different from the estimate of the effect  Very low certainty: We have very little confidence in the effect estimate: The true effect is likely to be substantially different from the estimate of effect  a. Due to moderate heterogeneity for the effects of grouped means, due to the size and characteristic differences between sample participants. However, we followed recommendations^1^ and used the random model to try to explain potential effect modifiers, excluding discrepant data through the lack of overlapping 95% CI and using subgroup analysis and/or meta-regression. Furthermore, we added in our analysis the range of uncertainty of the effect, described by the IP 95%.  b. We can consider a moderate imprecision of the estimated effect due to the null line being crossed by the CI and the range of CIs that we can consider relevant for some outcomes.  ^1^ Higgins JPT, Cochrane Collaboration. Cochrane handbook for systematic reviews of interventions. Second edition. ed. Wiley-Blackwell; 2019:xxviii, 694 pages. | | | | | | |  |
