# Supplementary material for: Effects of Cocoa Consumption on Cardiometabolic Risk Markers: Meta-Analysis of Randomized Controlled Trials
Source: Nutrients. 2024 Jun 18;16(12):1919. doi: 10.3390/nu16121919 (PMC11206597; doi:10.3390/nu16121919)
Supplement: Supplementary file 1 [file nutrients-16-01919-s001.zip › Supplementary Material File S5.docx]

**Supplementary Materials S5. Sensitivity Analyzes.**

Effects of Cocoa Consumption on Cardiometabolic Risk
Markers: Meta-Analysis of Randomized Controlled Trials

Tainah O. P. Arisi ^1^, Diego Silveira da Silva ^1^, Elana Stein ^1^, Camila Weschenfelder ^1^,
Patrícia Caetano de Oliveira ^1^, Aline Marcadenti ^1,2,3^, Alexandre Machado Lehnen ^1,^* and Gustavo Waclawovsky ^1^

^1^ Instituto de Cardiologia do Rio Grande do Sul/Fundação Universitária de Cardiologia,
Porto Alegre 90620-001, RS, Brazil; tainahortiz05@gmail.com (T.O.P.A.);
dieguitoef@hotmail.com (D.S.d.S.); elanast.nutricao@gmail.com (E.S.); camilawesche@gmail.com (C.W.);
fisio.patriciacaetano@gmail.com (P.C.d.O.); marcadenti@yahoo.com.br (A.M.); gwaclawovsky@gmail.com (G.W.)

^2^ Instituto de Pesquisa Hcor (IP-Hcor), Hcor 04005-909, São Paulo, SP, Brazil

^3^ Faculdade de Saúde Pública, Universidade de São Paulo (FSP-USP), São Paulo 01246-904, SP, Brazil

***** Correspondence: amlehnen@gmail.com; Tel.: +55-(51)-3230-3600 (ext. 3636/3757)

**Total body mass**

**
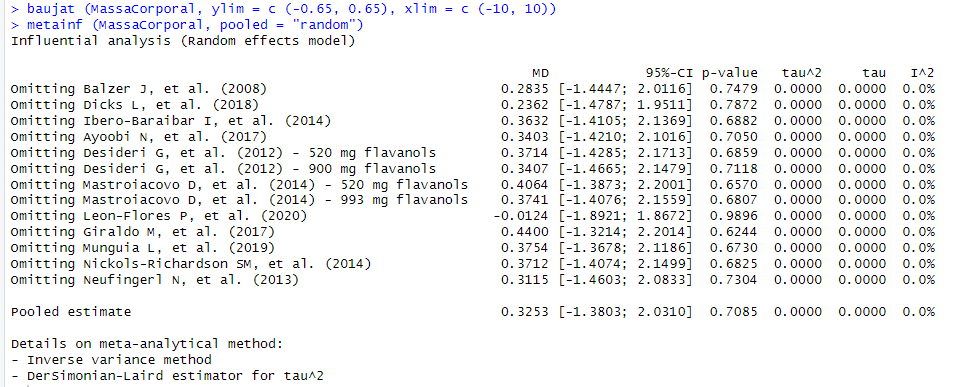
**

**BMI**

**
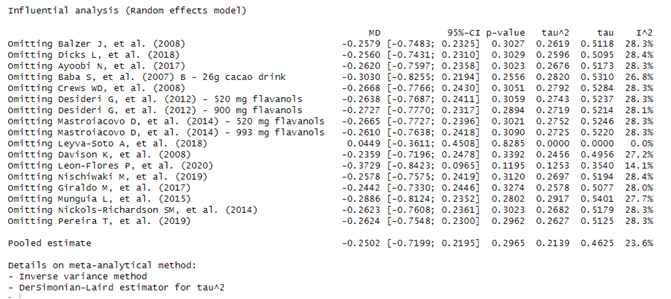
**

**Waist circumference**


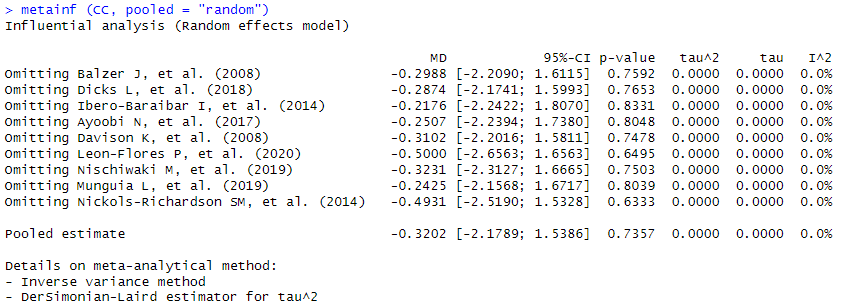


**Total cholesterol**

**
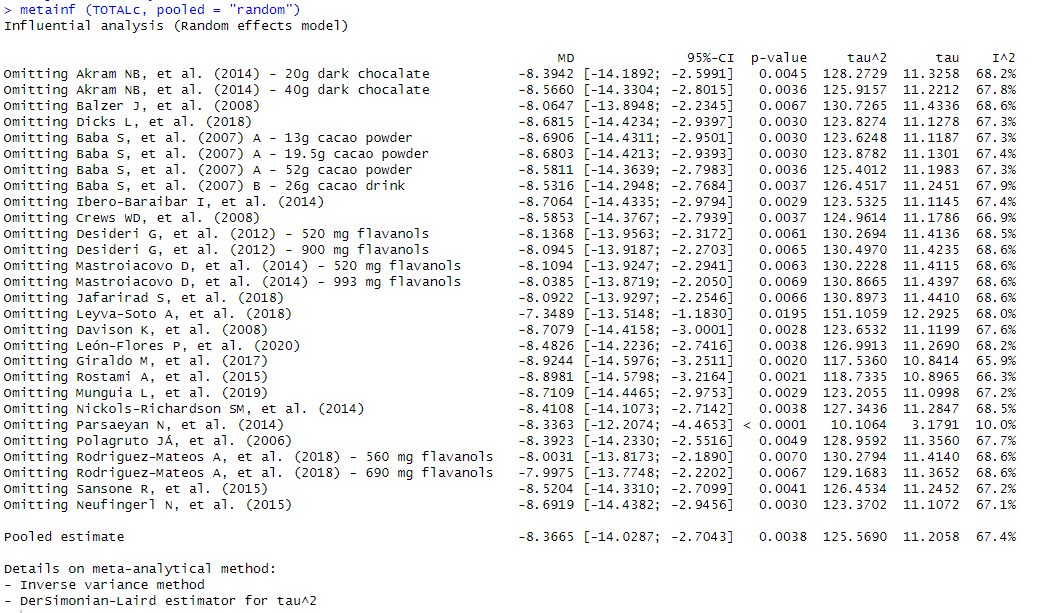
**

**LDL-c**


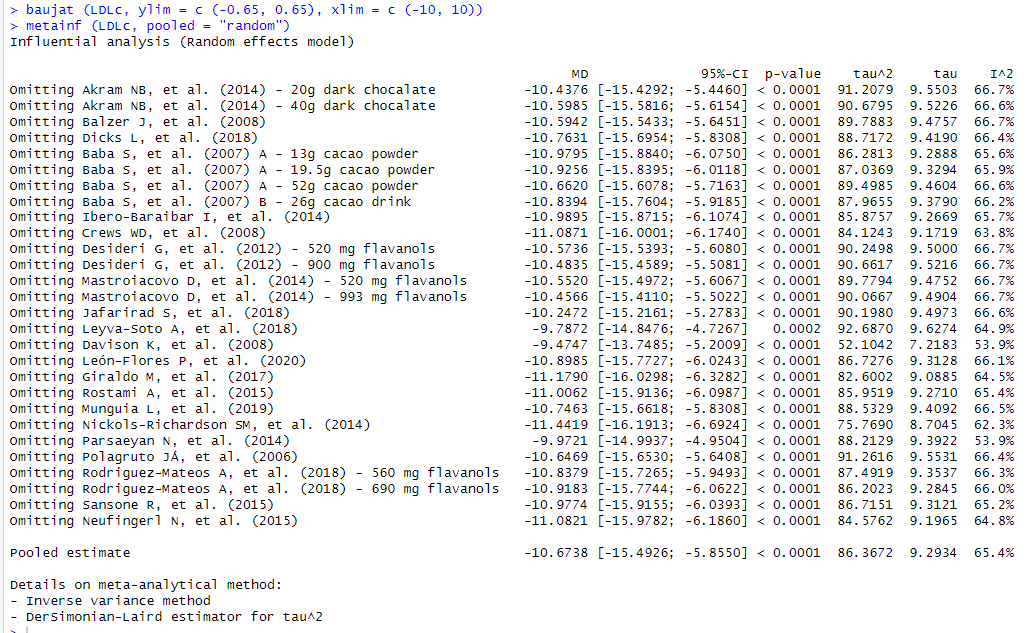


**Triglycerides**


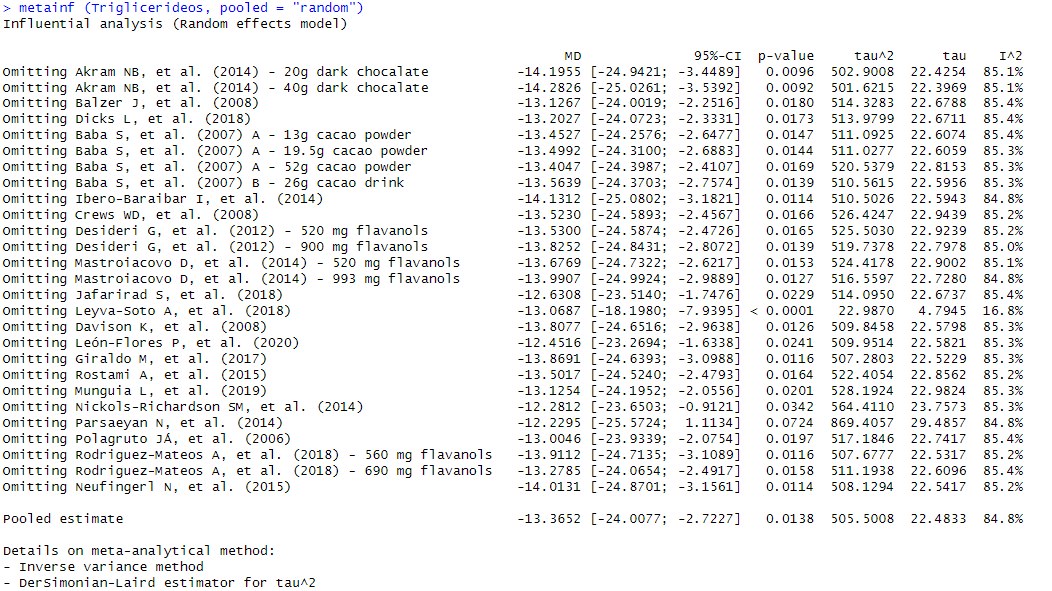


**HDL-c**

**
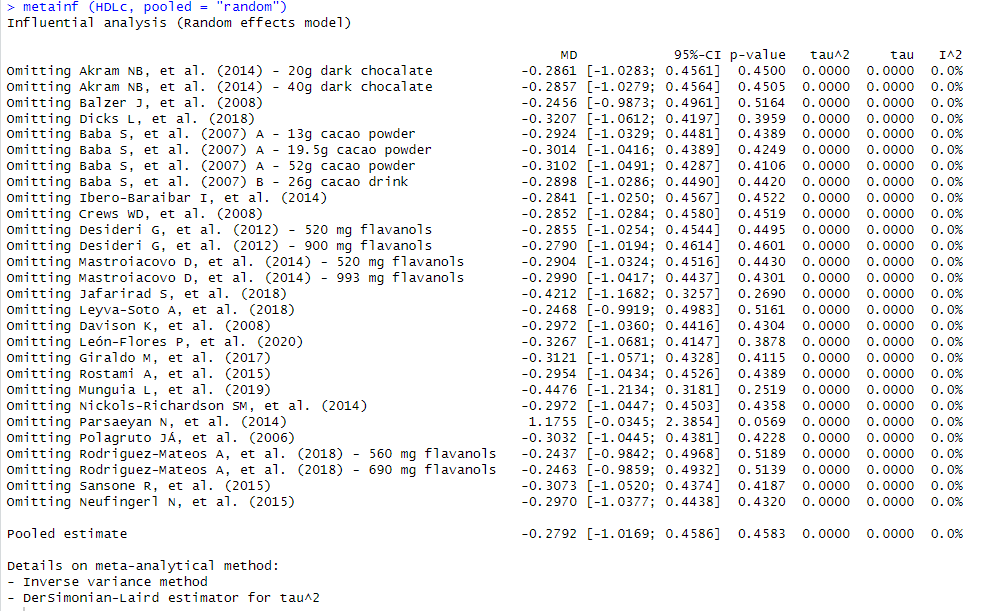
**

**Fasting blood glucose**

**
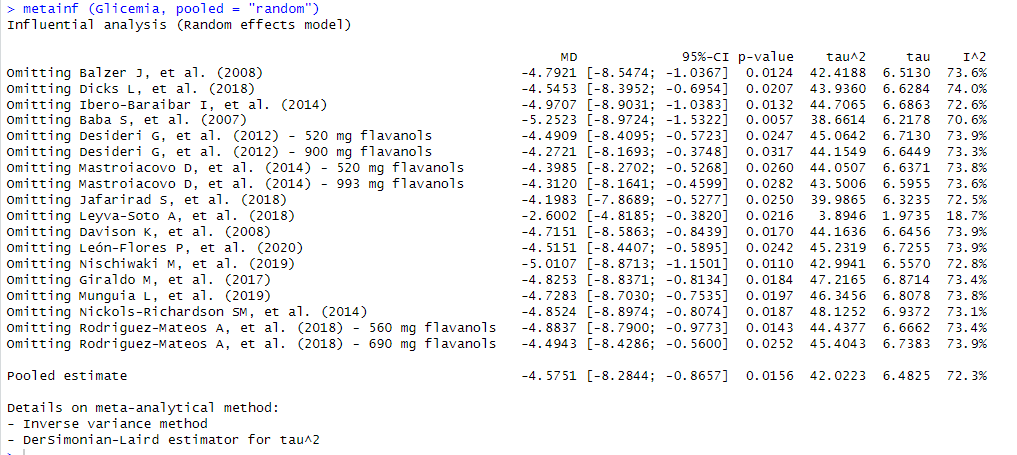
**

**HbA1c**


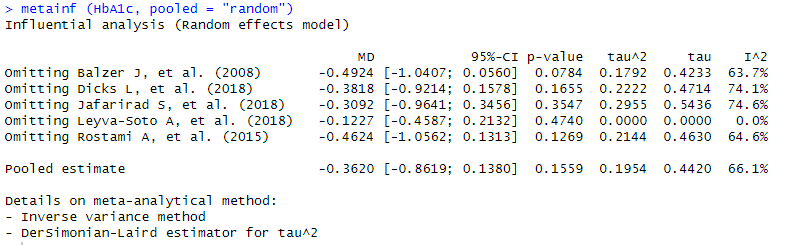


**SBP**

**
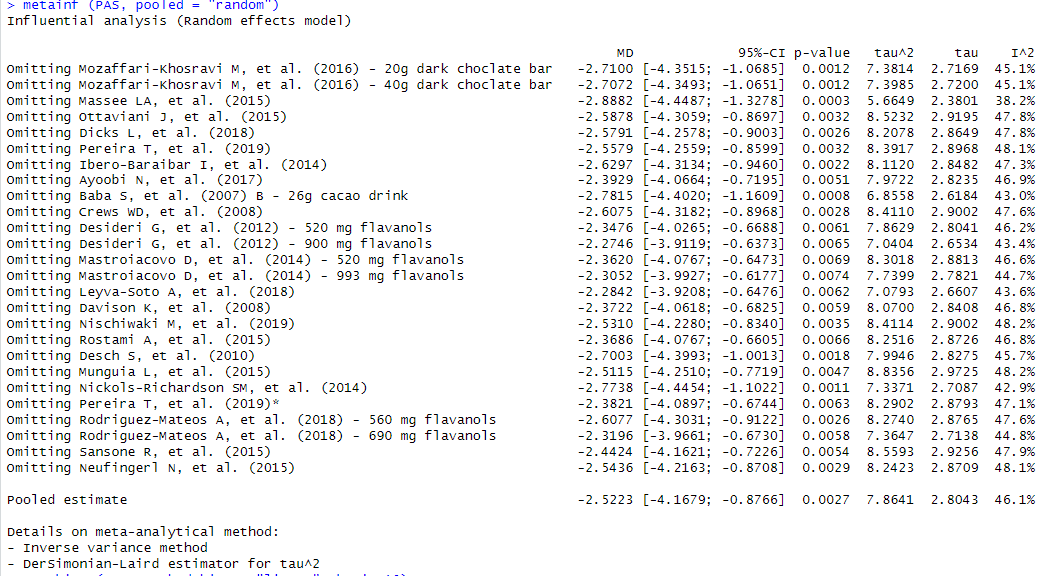
**

**DBP**

**
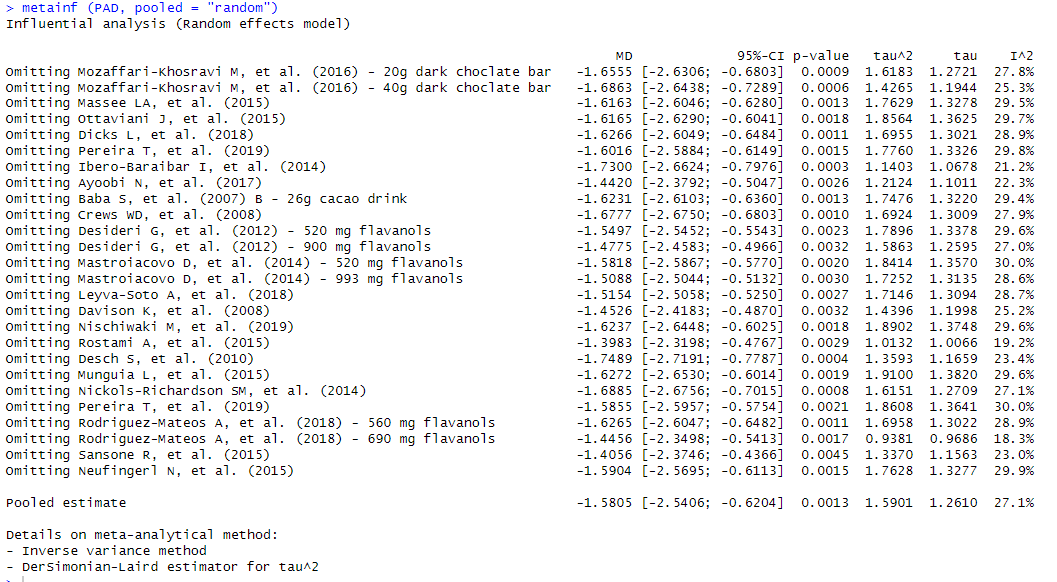
**
